# Geographical distribution of genetic diversity in *Secale* landrace and wild accessions

**DOI:** 10.1186/s12870-016-0710-y

**Published:** 2016-01-19

**Authors:** Jenny Hagenblad, Hugo R. Oliveira, Nils E. G. Forsberg, Matti W. Leino

**Affiliations:** IFM Biology, Linköping University, SE-581 83 Linköping, Sweden; CIBIO-Research Centre in Biodiversity and Genetic Resources, Campus Agrário de Vairão. R. Padre Armando Quintas, 4485-661 Vairão, Portugal; Nordiska Museet, Swedish Museum of Cultural History, SE-643 98 Julita, Sweden; Present Address: Faculty of Life Sciences, The University of Manchester. Manchester Institute of Biotechnology, 131 Princess Street, M1 7DN Manchester, UK

**Keywords:** Rye, Population structure, SNP, Ascertainment bias, Genetic variation, Phylogeography

## Abstract

**Background:**

Rye, *Secale cereale* L.*,* has historically been a crop of major importance and is still a key cereal in many parts of Europe. Single populations of cultivated rye have been shown to capture a large proportion of the genetic diversity present in the species, but the distribution of genetic diversity in subspecies and across geographical areas is largely unknown. Here we explore the structure of genetic diversity in landrace rye and relate it to that of wild and feral relatives.

**Results:**

A total of 567 SNPs were analysed in 434 individuals from 76 accessions of wild, feral and cultivated rye. Genetic diversity was highest in cultivated rye, slightly lower in feral rye taxa and significantly lower in the wild *S. strictum* Presl. and *S. africanum* Stapf. Evaluation of effects from ascertainment bias suggests underestimation of diversity primarily in *S. strictum* and *S. africanum*. Levels of ascertainment bias, STRUCTURE and principal component analyses all supported the proposed classification of *S. africanum* and *S. strictum* as a separate species from *S. cereale. S. afghanicum* (Vav.) Roshev*, S. ancestrale* Zhuk.*, S. dighoricum* (Vav.) Roshev*, S. segetale* (Zhuk.) Roshev and *S. vavilovii* Grossh. seemed, in contrast, to share the same gene pool as *S. cereale* and their genetic clustering was more dependent on geographical origin than taxonomic classification. *S. vavilovii* was found to be the most likely wild ancestor of cultivated rye. Among cultivated rye landraces from Europe, Asia and North Africa five geographically discrete genetic clusters were identified. These had only limited overlap with major agro-climatic zones. Slash-and-burn rye from the Finnmark area in Scandinavia formed a distinct cluster with little similarity to other landrace ryes. Regional studies of Northern and South-West Europe demonstrate different genetic distribution patterns as a result of varying cultivation intensity.

**Conclusions:**

With the exception of *S. strictum* and *S. africanum* different rye taxa share the majority of the genetic variation. Due to the vast sharing of genetic diversity within the *S. cereale* clade, ascertainment bias seems to be a lesser problem in rye than in predominantly selfing species. By exploiting within accession diversity geographic structure can be shown on a much finer scale than previously reported.

**Electronic supplementary material:**

The online version of this article (doi:10.1186/s12870-016-0710-y) contains supplementary material, which is available to authorized users.

## Background

Rye (*Secale cereale* L.) has the ability to thrive and to produce high yields also under adverse environmental conditions [1, 2]. It is unique amongst old-world graminoid cereals for being an out-breeder (wind cross-pollinated) and thus constitutes an important species for comparative studies in crop evolution. Turkey, Transcaucasia, Iran and Central Asia are believed to be centres of domestication of rye but it is still unclear which route rye followed as it was introduced into Europe: north of the Black and Caspian seas into central Europe (and from here to the Balkans) or along the Mediterranean route followed by the other Neolithic cereals [3]. Rye was long a staple crop in central and northern Europe and Russia, but has been cultivated to a much lesser extent in other parts of Europe. In Fennoscandia (Finland, Sweden, Norway and Denmark), rye became a dominant food crop in early Medieval times [4, 5]. Especially in Finland rye was a staple crop and the main produce in the slash-and-burn farming systems practiced until the early 20^th^ century [6]. During the 20^th^ century, rye cultivation in Europe, including Fennoscandia, declined and the worldwide rye production was 16.7 million tonnes in 2013, making it only the 24^th^ most produced crop [http://faostat3.fao.org].

Cultivated rye is a diploid annual grass. Different taxonomies have been proposed for the genus *Secale* [7–10]. Recent studies have been conducted using molecular markers such as rDNA-ITS [11], 5S-rDNA [12], AFLPs [13, 14] and SSRs [15], but the taxonomy of the genus remains inconclusive. The relationship between cultivated, weedy, feral and true wild forms is also elusive [16]. For its simplicity, in this paper we follow the Sencer and Hawkes [8] classification with cultivated rye classified as the species *Secale cereale* subsp. *cereale*. Within the *S. cereale* species some weedy forms are included (*ie:* subsp. *segetale, dighoricum, afghanicum* and *ancestrale*). These weedy forms, here called feral, occur as weeds in cereal fields, mostly in the Near East and Central Asia and are fully inter-fertile with cultivated rye [17].

Wild ryes related to cultivated rye include *S. vavilovii* (*ie*: *S. cereale* subsp. *vavilovii*), distributed throughout southwest Asia, and *S. strictum*, occurring throughout the Mediterranean Basin, Southwest Asia, Caucasus and Central Asia [8, 18]. These wild ryes, especially *S. vavilovii*, can hybridise with *S. cereale* [8]. It is still debated whether cultivated rye was domesticated from one or both of these two wild species [19]. In the most recent morphology-based taxonomy Frederiksen and Petersen [10] considered only three species: the annual wild *S. sylvestre*; the perennial wild *S. strictum* (= *S. montanum*) (with subspecies *strictum*, *africanum*, and *anatolicum*); and *S. cereale*, including cultivated and weedy rye and *vavilovii* as subspecies.

In many crops a large proportion of the genetic diversity of the species can be found in unimproved domesticated varieties, known as landraces. These can be defined as “dynamic population(s) of a cultivated plant that has historical origin, distinct identity and lacks formal crop improvement, as well as often being genetically diverse, locally adapted and associated with traditional farming systems” [20]. As a result of long lasting cultivation at their particular locations, landraces are likely to reflect the historical origins and the selection and adaptation processes affecting crops [21]. Thus, crop landraces are a superior material compared to elite breeds when it comes to the investigation of the distribution of genetic diversity resulting from crop evolutionary processes. Genebanks worldwide hold thousands of rye landrace accessions as well as seeds of feral and wild forms, preserving a vast diversity of agronomically relevant genes and traits.

The distribution of genetic diversity in different taxa of rye has been examined by various molecular marker systems. Persson & von Bothmer [22, 23] used isoenzymes and RAPDs to study landraces and cultivars from Northern Europe but found no clear structuring from geography or improvement status. Chikmawati et al. [13, 14] used AFLP and a worldwide collection of cultivated, wild and weedy rye. In their study, the wild and weedy rye was separated from the cultivated rye, but no geographic structure was found among the cultivated accessions. Recently, Bolibok-Bragoszewska et al. [24] used a massive pooling strategy and dominant DaRT markers to investigate genetic structuring among elite breeds, landraces and wild ryes. Taxon and breeding status was found to result in some genetic structuring whereas geography was mostly unrelated to genetic distribution. A common observation of the studies mentioned above is the high degree of variation found within groups and not among them. Consequently, to find geographic genetic structuring, high power in terms of marker number and individuals is needed. It is thus unfortunate that with the exception of the studies by Persson & von Bothmer [22, 23] within-accession diversity has not been explored in rye.

Lately single nucleotide polymorphisms (SNPs) have become the preferred molecular markers in crop genomics because of their high frequency across genomes and their amenability to cost-effective high-throughput assays [25]. SNPs are suitable markers for studying population structure and evolutionary processes in cereals and have been applied in the study of rice [26, 27], maize [28, 29], wheat [30, 31] and barley [32–34]. Recent efforts have resulted in SNP panels being developed also in rye [35–37] thereby allowing geographic structure and evolution to be investigated also in this outbreeding crop.

Our understanding of the evolution of domesticated plants in the Old World has mainly been based on self-pollinating plants with a long domestication history (e.g. wheat, rice, barley). In this paper we thus address the following questions: 1) How is genetic diversity distributed within and between populations of cultivated, wild and feral rye? 2) Does population structure corroborate the taxonomy of rye and from which wild species was rye domesticated? 3) Can we detect geographic structuring of genetic diversity in landrace rye and if so on which scale? For this purpose we genotyped a panel of 768 SNPs distributed throughout the rye genome in rye landraces and in feral and wild rye accessions.

## Materials and methods

### Plant material

A panel consisting of 468 individual plants belonging to 80 rye accessions from a broad geographic range including Europe, Morocco, Near East, Russia and Central Asia was assembled (Table 1; Additional file 1). Accessions were provided by the following genebanks with acronym, accession prefix and country indicated: United States Department of Agriculture Germplasm Resources Information Network (GRIN, PI, USA), Leibniz-Institut für Pflanzengenetik und Kulturpflanzenforschung (IPK, R, Germany), Nordic Genetic Resource Center (NordGen, NGB, Sweden), Instytut Hodowli i Aklimatyzacji Roślin (IHAR, PL, Poland), Institut National de Recherche Agronomique (INRA, INRA, France), Science and Advice for Scottish Agriculture (SASA, SASA, Scotland). One accession (KENO004) was collected ‘on farm’ in 2012 (Annika Michelsson, pers. com.). The panel included cultivated rye landraces, cultivated elite breeds (‘Imperial’, ‘Kungs II’ and ‘Petkus’), feral rye (*S. cereale* subsp. *afghanicum, ancestrale, dighoricum* and *segetale;* henceforth referred to by their sub-specific classification) and wild ryes (*S. strictum, S. africanum, S. vavilovii*) (Table 1 and Additional file 1). Accessions for which passport data regarding growth habit (winter vs spring) were lacking were test cultivated in a greenhouse. Accessions that flowered and produced ears within less than two months without vernalization were considered to be of spring habit, while those that had not flowered were considered to be of winter habit. DNA was extracted from young leaves of 6 individual plants of each accession using the DNeasy Plant Mini Kit (Qiagen, Hilden, Germany) or the E.Z.N.A^®^ Plant DNA kit (Omega Biotek Inc., Norcross, GA, US).

**Table 1:** Accessions used in this study: their type, taxonomical classification and geographical provenance

Type	Taxon	No. of accessions	No. of individuals	Provenance
Wild	*S. strictum, S. africanum, S. vavilovii*	8	44	Iran, Italy, South Africa, Turkey.
Feral	*S. cereale* subsp. *afghanicum, ancestrale, dighoricum, segetale*	17	97	Afghanistan, Azerbaijan, Pakistan, Russia, Spain Sweden, Turkey, Turkmenistan.
Cultivated Landraces	*S. cereale* subsp*. cereale*	48	275	Afghanistan, Austria, Belarus, Bosnia, Czech Republic, Finland, France, Germany, Greece, Hungary, Italy, Montenegro, Morocco, Norway, Poland, Portugal, Romania, Russia, Scotland, Spain, Sweden, Switzerland, Tajikistan, Turkey, Ukraine.
Cultivated elite breeds	*S. cereale* subsp*. cereale*	3	18	Germany, Sweden, USA.

### SNP genotyping

Genotyping was performed using the Illumina Golden Gate assay at the SNP&SEQ Technology Platform at Uppsala University, Sweden. A panel of 768 SNPs were genotyped following the service provider’s protocol. The SNPs assayed were chosen from a panel developed by Haseneyer et al. [36] (Additional file 2). Due to lack of mapping information at the time, SNPs were selected to represent different biological roles, as described in their annotation. Later obtained mapping information [38] for ~100 of the SNPs showed an even distribution among chromosomes. SNPs not fulfilling Illumina Golden Gate design recommendations were avoided. Results were analyzed using the software GenomeStudio 2011.1 (Illumina).

### Chloroplast SSR genotyping

The rye plants screened for the SNP panel were also genotyped with five chloroplast SSRs (cpSSRs) (Wct2, Wct12, Wct13, Wct15, Wct22), developed for *Lolium* and wheat but applicable to rye [39, 40]. The forward primer of the cpSSR markers was labelled with either of two fluorescent dyes: 6-FAM or HEX and PCR products were analysed on an ABI PRISM® 3730 DNA Analyser at NTNU (Trondheim, Norway). Chromatograms were analysed using GeneMapper® 3.7 software with alleles scored using the binning function.

### Data analysis

Accessions were grouped on the basis of taxon, biological type (*ie:* wild, feral and cultivated) and geographic provenance of cultivated landraces. The latter was based on the four agro-climatic zones proposed by Bouma [41]: Maritime, Mediterranean, Central and North East. Five accessions located outside of the region studied by Bouma were excluded from analyses of agro-climatic zones.

Allele frequencies and genetic diversity measures were calculated using PowerMarker 3.25 [42] and GenAlEx 6.5 [43]. These measures included expected (under Hardy-Weinberg equilibrium) and observed heterozygosity (H_E_ and H_O_), number of alleles and inbreeding coefficient (fixation index, F). Measures were calculated both within each accession, and across all accessions within the groups of taxon, biological type and agro-climatic zone respectively. To evaluate the effects of ascertainment bias genetic diversity was also calculated for haplotypes of length two to five SNPs. Based on mapping data [38] SNPs with a known mapping position were merged into haplotypes consisting of two to five neighbouring SNPs, which were then used for diversity calculations.

Pairwise genetic and geographic distances between accessions and pairwise F_ST_ between different groups as well as AMOVAs were calculated using GenAlEx 6.5, using 999 permutations for testing variance components. To investigate Isolation-by-Distance we used GenAlEx 6.5 to compute a Mantel test (using 999 permutations) for correlation between a genetic distance matrix and a geographic distance matrix for cultivated rye landraces. To assess whether rye cultivation spread in a slow stepwise fashion with few individuals migrating to new areas from previous populations starting in an original core area (assumed to be Turkey [14, 17]), we plotted the genetic diversity (H_E_) of each landrace against its distance to origin as well as latitude and longitude.

Population structure in our *Secale* accession panel was investigated using the Bayesian model-based approach implemented in the STRUCTURE 2.3.4 software [44]. The program was run with values of *K* ranging from 1 to 12, with 20,000 burn-in iterations and 50,000 MCMCs, with 10 independent runs for each K, using the admixture model with correlated allele frequencies. The most likely value of K was evaluated from the CLUMPP H' values [45] and *ΔK* according to the Evanno et al. [46] method. STRUCTURE was run for the complete dataset and for subsets of accessions to infer structure within taxonomic groups, within clusters detected during the analysis of the full data set and within geographical areas. R 3.0.2 [47] was used for evaluating cpSSR markers for population structure with discriminant analysis of principal components (DAPC) using the *adegenet* package [48]. Clusters for the analysis of cultivated rye were mapped in ArcGIS 10.0 (ESRA).

Principal Component Analysis (PCA) was also computed with the R environment for statistical computing for the complete accession panel and for different subsets of cultivated rye. Computation of PCA was based on a matrix of allele frequencies for each accession at each locus. The data from the PCA was further used to generate a relative measure of genetic relatedness within accessions, PC dispersion [34]. This measure, calculated in R, utilizes mean pair-wise distances in the PC-space between individuals belonging to the same accessions. Information from all principal components was included as multidimensional coordinates.

To compare the effects of analysing genetic diversity based on multiple samples of the same accessions with that based on pooled samples we carried out *in silico* pooling of our accessions. In the *in silico* pooling we assumed that each individual rye extraction contributed equally to the genotype scoring of the pool, which would be the ideal case if equal molar amounts of DNA were added from each extraction. We then chose an *ad hoc* cut-off point of 0.75 to create an interpretation reflecting the SNP scoring procedure and limiting the loss of information. Each accession was assigned a heterozygous genotype if the allele frequency of the more common allele was less than 0.75. If the more common allele was present in the accession at higher frequencies than 0.75 the accession was assigned a homozygous genotype. The resulting accession genotypes were used for diversity and structure analyses as described above.

## Results

### Genotyping success

We genotyped 468 individuals from 80 accessions for a total of 768 SNP markers. Although we aimed to analyse six individuals per accession, in some instances, due to low DNA quality or to make room for positive and negative controls in 96-well plates, some samples had to be excluded and only five individuals were analysed for some accessions. Of the 768 SNPs assayed 134 failed to produce genotyping results. An additional 32 markers failed in more than 50 % of the individuals screened and 35 proved to be monomorphic. All these 201 markers were thus removed from the dataset before further analysis. Of the 468 individuals initially screened, 11 failed to produce reliable calls for any marker and 5 had too many missing data points and were removed before further analysis. Two accessions were also removed for containing data from less than four individuals. Additionally, two *S. strictum* accessions (PI 240285 and PI 531829, 12 individuals) were excluded after doubts about their taxonomic classification (see further below). After the exclusion of markers and individuals, a final dataset consisting of 567 SNPs screened in 434 individual plants belonging to 76 accessions were used for further analysis.

Among the 567 SNPs analysed for the 434 individuals in the final dataset a 95 % genotype scoring success was obtained. Although initially developed for cultivated rye elite varieties, the SNP panel worked efficiently for all taxa, with *S. afghanicum* and *S. segetale* having the lowest proportion of missing data (2.73 % and 3.62 % respectively) and the wild ryes *S. strictum* and *S. africanum* having the highest (8.82 % and 5.76 % respectively).

### Genetic diversity

Both alleles of most of the biallelic markers could be found in all three groups of biological types, wild (average 1.974 alleles per marker), feral (average 1.993 alleles per marker) and cultivated (both alleles found in all markers) as well as in the different taxa (Na in Table 2). Looking within accessions, however, monomorphic markers were more common in the wild and feral accessions than in cultivated accessions. With the exception of *S. africanum* minor allele frequencies were fairly evenly distributed (Additional file 3). Total genetic diversity H_E_ was highest in cultivated rye and lowest in wild (Table 2). The taxon with the highest H_E_ was *S. cereale*, likely an effect of ascertainment bias during the SNP discovery (see below), followed by *S. vavilovii* and *S. segetale. S. strictum* and *S. africanum* had the lowest H_E_ (Table 2). Differences in total genetic diversity between geographical groups of cultivated rye were very small. Inbreeding coefficients (F) were in general low as could be expected from an outcrossing species (Table 2). However, notably, some taxa (e.g. *S. dighoricum*) have higher inbreeding coefficients than others, possibly indicating more limited geneflow within this taxon or higher rates of self-pollination. Likewise, among geographical groups of cultivated landraces, inbreeding coefficients are somewhat higher in the Central and Mediterranean groups than in the North East and Maritime groups (Table 2).

**Table 2:** Summary of genetic diversity measures for the complete accession panel and selected subgroups based on 567 polymorphic SNPs. Both within accession averages and total diversity within groups are shown as well as diversity upon sample pooling *in silico. N*: sample size – number of accessions (number of individuals within brackets); *Na*: number of alleles; H_O_: Observed Heterozygosity; H_E_: Expected Heterozygosity; *F*: Fixation Index

Group		*N*	*Na*	*H*_*O*_	*H*_*E*_	*F*
Biological type						
Wild	Within acc.	8 (44)	1.526	0.214	0.187	−0.151
	*In silico* pooled		1.825	0.240	0.256	0.028
	Total		1.974	0.224	0.286	0.178
Feral	Within acc.	17 (97)	1.675	0.257	0.241	−0.078
	*In silico* pooled		1.952	0.309	0.294	−0.032
	Total		1.993	0.259	0.329	0.206
Cultivated	Within acc.	51 (290)	1.777	0.316	0.283	−0.114
	*In silico* pooled		1.977	0.375	0.309	−0.162
	Total		2.000	0.313	0.347	0.096
Taxon						
*S. africanum*	Within acc.	1 (6)	1.568	0.215	0.188	−0.131
	*In silico* pooled		1.220	0.220	0.110	−1.000
	Total		1.568	0.215	0.188	−0.131
*S. strictum*	Within acc.	4 (20)	1.317	0.134	0.114	−0.172
	*In silico* pooled		1.429	0.158	0.144	−0.113
	Total		1.568	0.135	0.152	0.083
*S. vavilovii*	Within acc.	3 (18)	1.790	0.321	0.284	−0.128
	*In silico* pooled		1.686	0.356	0.262	−0.325
	Total		1.949	0.322	0.327	0.009
*S. afghanicum*	Within acc.	4 (21)	1.666	0.254	0.242	−0.067
	*In silico* pooled		1.693	0.312	0.250	−0.223
	Total		1.903	0.254	0.297	0.124
*S. ancestrale*	Within acc.	5 (30)	1.707	0.267	0.243	−0.100
	*In silico* pooled		1.725	0.292	0.244	−0.177
	Total		1.963	0.268	0.301	0.092
*S. dighoricum*	Within acc.	4 (23)	1.639	0.231	0.229	−0.026
	*In silico* pooled		1.771	0.311	0.276	−0.129
	Total		1.938	0.235	0.310	0.218
*S. segetale*	Within acc.	4 (23)	1.680	0.274	0.249	−0.113
	*In silico* pooled		1.741	0.324	0.274	−0.166
	Total		1.944	0.278	0.322	0.118
*S. cereale*	Within acc.	51 (290)	1.777	0.316	0.283	−0.114
	*In silico* pooled		1.977	0.375	0.309	−0.162
	Total		2.000	0.313	0.347	0.096
Geographical provenance^a^						
Central	Within acc.	6 (35)	1.738	0.304	0.271	−0.123
	*In silico* pooled		1.838	0.374	0.299	−0.217
	Total		1.981	0.304	0.338	0.088
Maritime	Within acc.	14 (79)	1.780	0.329	0.285	−0.144
	*In silico* pooled		1.910	0.392	0.307	−0.225
	Total		1.989	0.327	0.344	0.047
Mediterranean	Within acc.	15 (86)	1.785	0.315	0.285	−0.103
	*In silico* pooled		1.924	0.380	0.301	−0.209
	Total		1.993	0.314	0.343	0.084
North East	Within acc.	8 (43)	1.814	0.322	0.294	−0.095
	*In silico* pooled		1.788	0.374	0.270	−0.315
	Total		1.988	0.321	0.333	0.032

^a^For landrace rye only. Based on Bouma’s [41] proposed agro-climatic zones

Average within-accession diversity for groups was just somewhat lower than total diversity, showing that most diversity is captured within accessions, and to a lesser extent distributed between accessions. Differences in average within-accession H_E_ are statistically significant both comparing biological type and taxa (two-way ANOVA, both *P* < 0.001). Among cultivated rye landraces from different regions, differences in genetic diversity, H_E_, were small and non-significant (one-way ANOVA, *P* = 0.16). *In silico* pooling of accession genotypes showed that a genotyping strategy of pooled individuals would have in general captured between 80 and 94 % of the genetic diversity of the accessions (Table 2). No significant differences in inbreeding coefficients (F) for accessions were found among biological types (*P* = 0.06), taxa (*P* = 0.54) or geographical regions (*P* = 0.44). Looking at single accessions, within-accession diversity was lowest in the two *S. strictums* PI 401405 (0.092) and PI 401399 (0.090) while the highest within-accession diversity was detected in the Swedish landrace NGB21083 (0.313) and the *S. segetale* accession PI 326284 (0.314) (Additional file 1). Within-accession H_E_ was not significantly lower in commercial cultivars than in landraces (*t*-test, *P* = 0.56).

**Fig. 1:**
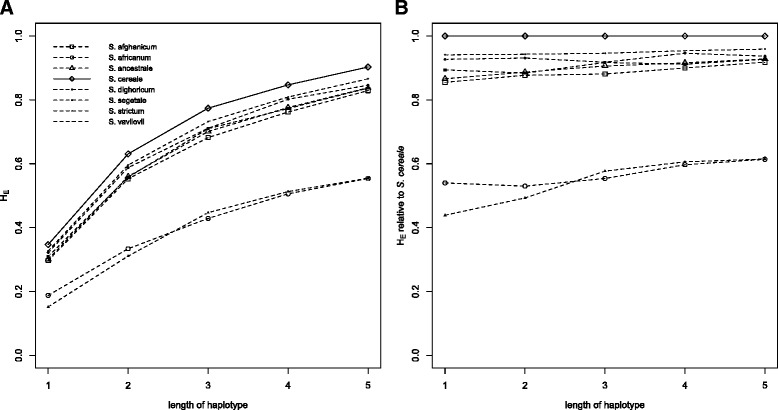
Genetic diversity of the different taxa studied for individual SNPs and neighbouring SNPs merged into haplotypes of length 2 – 5 SNPs. **a** Genetic diversity (H_E_). **b**) Genetic diversity relative to the diversity of *S. cereale*

In conclusion we find high diversity levels within single accessions and increasing diversity levels going from wild to feral to domesticated rye. Ascertainment bias could be a possible cause for the differences in diversity between different biological types. When the distribution of minor allele frequencies of the marker were compared with the distribution expected under neutrality the presence of ascertainment bias was clear from the deficit of low frequency alleles and the excess of higher frequency alleles (Additional file 4). However, also the wild and feral rye, not part of the material used to ascertain the SNPs showed a clear deficit of lower frequency alleles suggesting that the effects of ascertainment bias were not substantially different between the three types of material (Additional file 4). To further evaluate the effects of ascertainment bias on the estimate of genetic diversity we merged SNPs that had been mapped to neighbouring positions into haplotypes consisting of two to five neighbouring SNPs. Such merging of SNPs into haplotypes has previously been shown to alleviate the effects of ascertainment bias [49]. Merging SNPs into increasingly long haplotypes had little effect on the relative ranking of the different rye taxa and *S. strictum* and *S. africanum* were still the least diverse taxa when merging SNPs into 5-SNP haplotypes (Fig. 1a). With large amounts of ascertainment bias the relative diversity of the different taxa should become more similar with increasing haplotype length. Compared to the diversity in *S. cereale* most taxa showed a limited such increase (less than 10 % for *S. afghanicum, S. ancestrale, S. dighoricum, S. segetale* and *S. vavilovii*). *S. africanum* (12 % increase) and *S. strictum* (28 % increase) did, however, show a clear increase in diversity relative to *S. cereale* (Fig. 1b).

The distribution of genetic diversity between and within different taxa and biological types was analysed using AMOVA (Table 3). As ascertainment bias is likely to bias the partitioning of molecular variation [50] *S. africanum* and *S. strictum* accessions were excluded from the AMOVA. The AMOVA results confirmed that diversity was primarily found within accessions. Among taxa, 3 % of the diversity was found, among types (wild vs feral vs cultivated) only 1 % of the diversity and among cultivated rye from different agro-climatic zones, 1 % of the diversity was found between regions. The large proportion of diversity found within accessions for all three types of groupings suggests high gene flow between different accessions, reflecting the wind-pollinated reproduction of rye.

**Table 3:** Analysis of molecular variance (AMOVA) for 405 individuals, 71 accessions, six taxa, three biological types and four geographic regions

Group	df	SS	Variance component	% of the total variance
Biological type^a^				
Among Type	2	1022.300	1.442	1 %
Among Accessions	68	17417.886	14.576	14 %
Within Accessions	739	66529.167	90.026	85 %
Total	809	84969.353	106.044	100 %
Taxon^a^				
Among Taxa	5	2278.843	2.684	3 %
Among Accessions	65	16160.593	13.919	13 %
Within Accessions	739	66529.167	90.026	84 %
Total	809	84968.602	106.629	100 %
Geographical provenance^b^				
Among Regions	3	1012.993	0.977	1 %
Among Accessions	39	8691.992	11.403	11 %
Within Accessions	443	41691.525	94.112	88 %
Total	485	51396.510	106.492	100 %

df: degrees of freedom; SS: sum of squares

^a^Excluding *S. africanum* and *S. strictum* accessions

^b^For landrace rye only. Based on Bouma’s [41] proposed agro-climatic zones

### Population structure

We investigated our data for genetic structure by initially running STRUCTURE for the full final data set. The values of *∆K* and CLUMPP H' indicated *K =* 2, 3 and 9 as the models best describing genetic structure in our rye accessions (Additional file 5). From the Q-matrix plots the presence of admixture could be seen, as different individuals within the same accession sometimes showed membership to several different clusters (Additional file 6). The first clusters STRUCTURE detected (*K* = 2) were one comprising some of the wild *S. strictum* accessions plus the accession of *S. africanum* (dark green in Additional file 6), and a second containing all cultivated and feral rye accessions as well as the wild rye *S. vavilovii*. The *S. strictum - africanum* cluster remained intact while increasing *K* to the value of 12 (Additional file 6). At *K* = 2 we noted that two accessions labelled as *S. strictum* (PI 240285 and PI 531829) did not cluster with *S. africanum* and the other *S. strictum* accessions but rather with the remaining rye. This observation and inspection of spike morphology, not showing the disarticulating rachis significant for *S. strictum* [10], cast doubts about them being *de facto S. strictum*. We therefore decided to exclude them from all analyses where taxonomic status was relevant.

At *K =3*, the *S. strictum* and *S. africanum* cluster remained intact (Additional file 6). The other cluster was split in two with the new clusters mainly reflecting a geographical division between accessions from Asia and Europe. *S. cerale* landraces from Asia (left side of *S. cereale* panel) showed the highest similarity to most of the feral ryes originating from the same region. Landraces from Western Europe also showed a degree of clustering with these ryes, while landraces from Italy, Eastern and Northern Europe clustered together at a high degree.

**Fig. 2:**
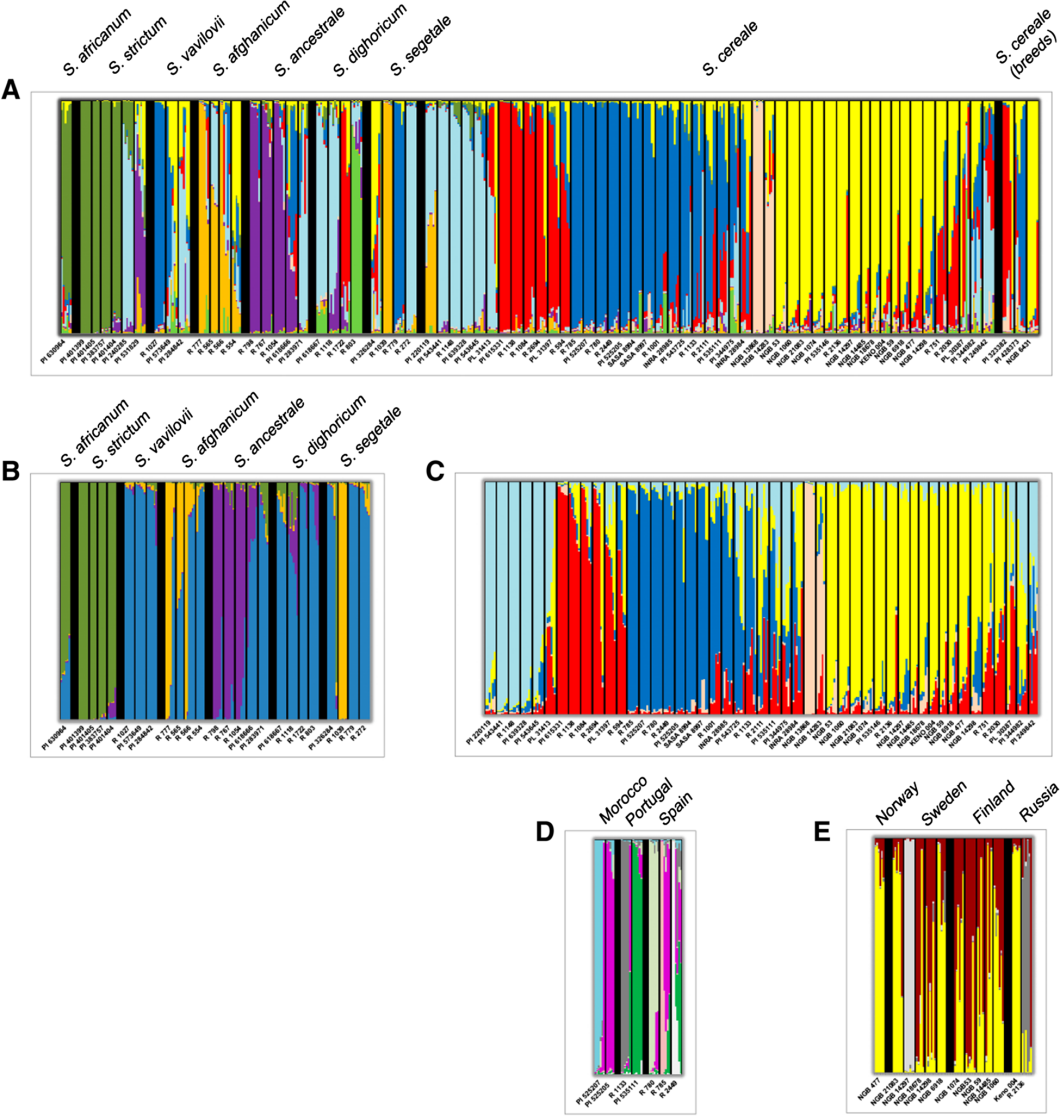
Clustering of rye individuals based on multilocus analysis using STRUCTURE. Accessions are organised by taxa. Each individual is depicted by a vertical line segmented into *K* coloured sections. The length of each section is proportional to the estimated membership coefficient (Q) of the individual accession to each one of the *K* clusters. Thin black vertical lines separate different accessions and thick ones separate different taxa. Labels on the x axis indicate accession numbers. **a** *K*= 9 model for the complete set of accessions, including the two *S. strictum* accessions that were later removed from the accession panel (PI 240285 and PI 531829). **b**) *K* = 4 model for the wild and feral rye accessions. **c**) *K* = 5 model for cultivated rye landraces. **d**) *K* = 7 model for the Southern set of Moroccan, Portuguese and Spanish landraces. **e**) *K* = 4 model for the Northern set of Fennoscandian and Russian landraces

When the model *K =* 9 is considered, five clusters were observed within the cultivated rye (Fig. 2a). These five clusters largely reflected geographic origin. One cluster consisted mainly of accessions from Northern Europe (yellow in Fig. 2a), a second cluster (dark blue) included cultivated rye accessions mostly from the west but also from Switzerland and Turkey as well as an accession of *S. vavilovii* from Italy, a third cluster is prevalent in Central Europe (red cluster). Accessions from the Balkans and Asia were found a fourth cluster (turquoise). The last cluster (pink) consisted of two accessions from a limited area, Finnmarken, on the border between Norway and Sweden. At low levels of *K* these individuals clustered with other Fennoscandian and Eastern European accessions. However, already at *K* = 5 they were beginning to separate from other Fennoscandian ryes and at *K* = 7 they were forming a cluster distinct from all other ryes (Additional file 6).

At the *K* = 9 level the accessions in some of the feral ryes, such as *S. ancestrale* and *S. afghanicum* showed fairly consistent clustering while others such as *S. vavilovii, S. dighoricum* and *S. segetale* showed a mixed clustering. It is worth noting that the accessions of *S. ancestrale* and *S. afghanicum* had a much less widespread origin than the accessions in the other three taxa. For example, the *S. ancestrale* accession PI 283971 with an origin assigned to Algeria clustered apart from the remaining *S. ancestrale* accessions with origins in Turkey and Turkmenistan. The three breeds included in the analysis did not cluster separately from landraces, but were split on different cluster groups, partly reflecting their geographical origin.

In order to confirm the general clustering and investigate substructure within the clusters detected we ran STRUCTURE with different subsets of accessions. When STRUCTURE was run excluding the *S. strictum* and *S. africanum* accessions the population structure of the remaining accessions was maintained as in the full set of accessions (data not shown). Analysis of only wild and feral accessions had the highest support for *K* = 4 (though with high support also for *K *= 2 and 3) (Additional file 5, Fig. 2b). At this level *S. africanum* and *S. strictum* clustered separately from *S. vavilovii* and the feral ryes. The other ryes all had accessions clustering together (dark blue in Fig. 2b) but with some accessions among *S. afghanicum* and *S. ancestrale* showing clustering similar to the one detected in the full dataset at *K* = 9. The geographic clustering observed among feral rye accessions in the full dataset was less evident when the structuring could not be anchored to the one among domesticated *S. cereale*. However, the geographically distant *S. segetale* R 1039 from Pakistan clustered with some of the *S. afghanicum* (only growing in Afghanistan) accessions rather than with the remaining *S. segetale*.

**Fig. 3:**
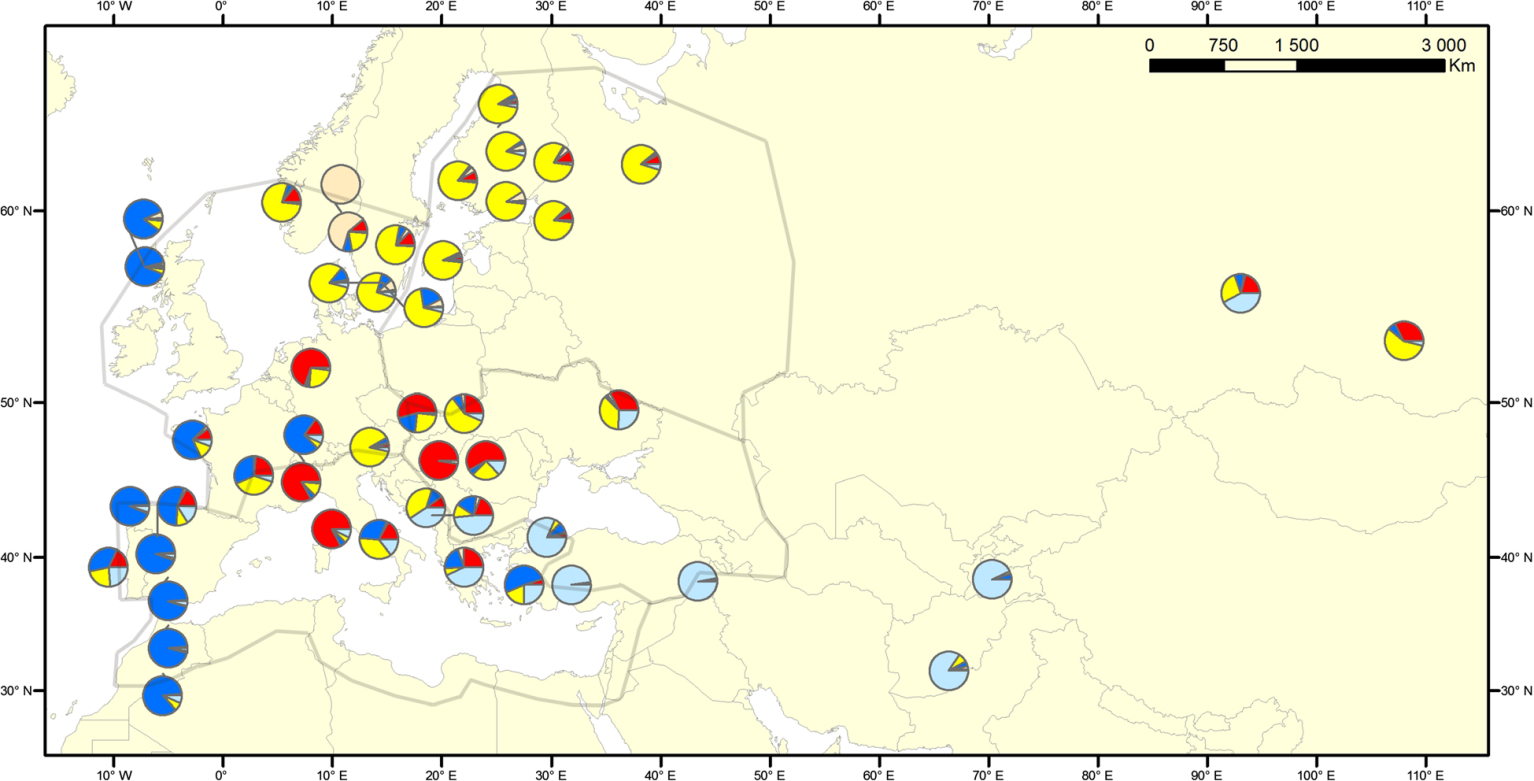
Geographical distribution of cultivated rye landraces clusters according to the *K* = 5 model in STRUCTURE. Each landrace is depicted as a pie chart with the proportional membership of its alleles to each one of the five clusters. Shaded areas represent the borders of Bouma’s [41] agro-climatic zones

When only cultivated landrace rye was analysed both *∆K* and CLUMPP H' values suggested *K* = 2 and *K* = 5 as the models with the highest support (Additional file 5). At *K* = 5 the main clusters observed agreed with the ones detected for the complete data set at *K* = 9 (Fig. 2c). The genetic structure detected was clearly geographically distributed, but showed limited overlap with the major agro-climatic zones proposed by Bouma [41] (Fig. 3). For example Southern Scandinavian accessions clustered with North Eastern accessions rather than maritime ones as suggested by its agro-climate. Additionally, Iberian and North African accessions showed little clustering with other accessions from the Maritime zone. We noted that accessions primarily belonging to the blue cluster in Western Europe and North Africa have spring habit and accessions belonging to the yellow and pink cluster in Northern Europe have winter growth habit. The other clusters, with accessions from Central Europe and the Mediterranean include both spring and winter types.

**Fig. 4:**
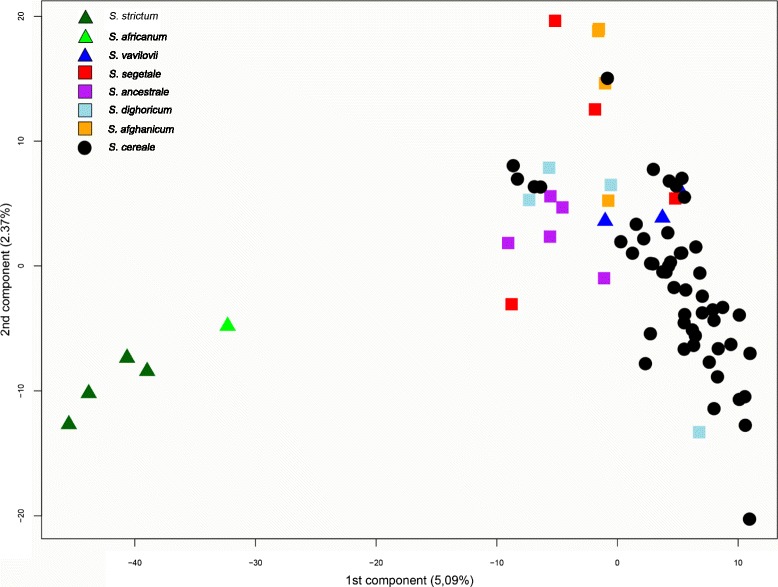
Plot of the 1^st^ and 2^nd^ components of a PCA analysis of the full data set based on the accession allele frequencies at 567 polymorphic SNP markers. Each point is an accession coloured according to the taxon it is classified as in its passport data

**Fig. 5:**
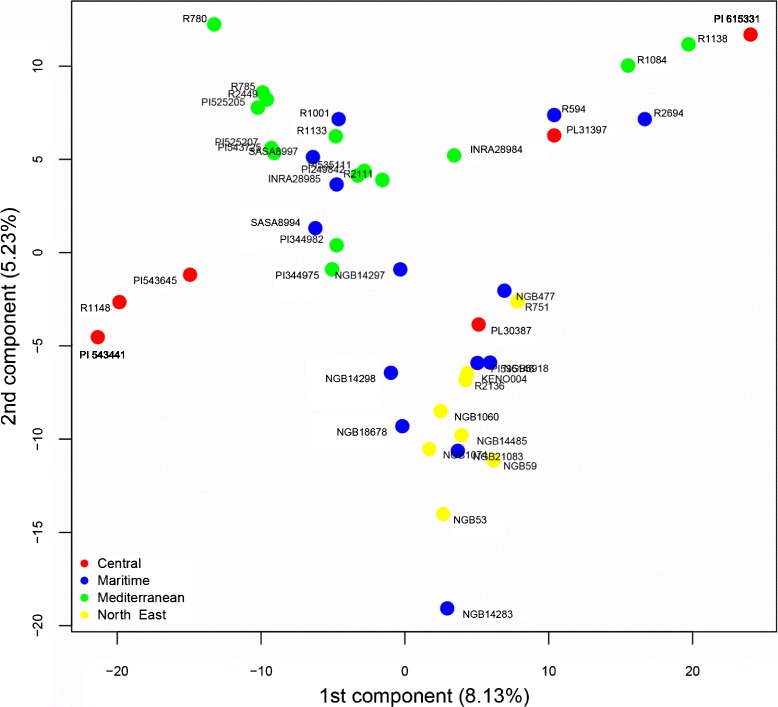
Plot of the 1^st^ and 2^nd^ components of a PC analysis of the rye landrace accession panel based on the allele frequencies of 567 polymorphic SNP markers. Each point is an accession coloured according to the agro-climatic zone it originates from, as defined by Bouma [41]

PCA confirmed that the *S. africanum* and the *S. strictum* accessions differed genetically from both the feral and cultivated rye (bottom-left quadrant in Fig. 4). PC1 showed a very clear distinction between the *S. africanum* - *S. strictum* and the *S. cereale* subspecies. In the cluster of *S. cereale* subspecies, *S. ancestrale* showed the clearest grouping whereas other subspecies proved to be more genetically diverse (Fig. 4). Cultivated rye accessions from areas with rye growing feral tended to be located close to the feral ryes rather than other cultivated rye. For example, accessions R 272 and R 1039 (*S. segetale*) and R 566, R 565 and R 777 (*S. afghanicum*) clustered around PI 220119 (landrace from Afghanistan, top-right quadrant). Accessions R 779 (*S. segetale)* from Spain and R 1027 (*S. vavilovii*) from Italy clustered closely with the cultivated Spanish landraces R 2449, R 785 and R 780 and the Moroccan landraces PI 525205 and PI 525207 (top-right quadrant) (Fig. 4). Focusing on the cultivated rye only, there was some agro-climatic clustering based on the zones of Bouma et al. [41], but with clear overlap and outliers (Fig. 5), as observed in the STRUCTURE analysis (Fig. 3). The North East group was clearly separated from the Mediterranean and Central group, but overlapped the Maritime group. However, all accessions in the Maritime group clustering with the North East group had Scandinavian origin.

We also calculated PC dispersion as a measure of the within-accession spread of individuals in the PC space (Additional file 1). Wild ryes in general showed less dispersion, that is were more homogenous, than both feral and cultivated rye (two-way ANOVA, *P* < 0.001) but there was no difference between feral and cultivated ryes. Among taxa, *S. strictum* accessions had lower PC dispersion than *S. cereale* accessions (*P* < 0.05) and *S. vavilovii* had higher PC dispersion than *S. ancestrale* (*P* < 0.05), *S. cereale* (*P* < 0.01) and *S. strictum* (*P* < 0.01), but no other groups of ryes differed in PC dispersion. The landrace accession NGB477 had a PC dispersion clearly lower than all other *S. cereale* accessions. Interestingly, the *S. africanum* accession that clustered together with *S. strictum* in the STRUCTURE analysis did not show a deflated PC dispersion.

From the PC dispersion measures, some *S. cereale* accessions showed inflated variance. This drew our attention to a few individuals that were genetically identical or highly similar, thus reducing the perceived genetic diversity of those accessions. Removing these individuals had no statistically significant effect on neither genetic diversity indices, pairwise F_ST_ or genetic distances (all *P *> 0.05, two-tailed *t*-test). We thus concluded that the presence of highly similar or identical individuals had a negligible effect on diversity measures.

Pairwise F_ST_ values were calculated between all pairs of taxa (Table 4). The highest F_ST_ was observed between *S. strictum* and *S. afghanicum* (0.298) and the lowest between *S. vavilovii* and *S. cereale* (0.016). The same taxa also had the highest and lowest pairwise genetic distances (0.154 and 0.017 respectively) (Table 4). It should be noted, however, that amongst our three *S. vavilovii* accessions, two originated in Europe. Looking at pairs of accessions the F_ST_ values ranged from 0.044 in a within *S. cereale* comparison to 0.329 in a comparison between a *S. segetale* and a *S. strictum* accession (Additional file 7). In general, comparisons including *S. strictum* accessions showed high F_ST_ values when compared with other accessions including other *S. strictum* accessions (Additional file 7). Additionally, the rye accessions from Finnmarken (NGB13868 and NGB14283) showed elevated F_ST_ values with all other accessions, regardless of taxon, compared to other landrace ryes.

**Table 4:** Pairwise F_ST_ values (below diagonal) and Nei’s pairwise genetic distance (above diagonal) between different taxons based on the allele frequency data for 567 polymorphic SNP markers. For the F_ST_ values, probability (P rand > = data), based on 999 pairwise population permutations, is < 0.001 except ^†^ *P* = 0.003 and ^‡^ *P* = 0.006. Correlation between the two matrices is 0.9697

	*S.afghanicum*	*S.africanum*	*S.ancestrale*	*S.cereale*	*S.dighoricum*	*S.segetale*	*S.strictum*	*S.vavilovii*
*S.afghanicum*	-	0.129	0.055	0.036	0.052	0.032	0.154	0.043
*S.africanum*	0.208	-	0.110	0.124	0.119	0.116	0.051	0.131
*S.ancestrale*	0.086	0.176	-	0.032	0.040	0.043	0.123	0.044
*S.cereale*	0.051	0.171	0.049	-	0.029	0.021	0.145	0.017
*S.dighoricum*	0.073	0.179	0.057	0.040	-	0.039	0.135	0.042
*S.segetale*	0.041	0.176	0.062	0.025	0.048	-	0.134	0.029
*S.strictum*	0.298	0.133	0.250	0.228	0.262	0.260	-	0.151
*S.vavilovii*	0.058	0.199	0.061	0.016^‡^	0.052	0.028^†^	0.286	-

To investigate Isolation-by-Distance we compared the genetic and geographic distances between cultivated rye landraces. No correlation between genetic and geographic distances was found (R = 0.179; *P* = 0.067) (Additional file 8). We also plotted genetic diversity against latitude and longitude respectively to see if adaptation to new environments or genetic bottlenecks during spread resulted in populations with reduced diversity. We observed no correlation between genetic diversity and longitude (R = 0.089; *P* = 0.273) (Additional file 9), but a significant correlation between genetic diversity and latitude (R = 0.387; *P* < 0.01). Landraces at higher latitudes, contrary to our expectations, proved to be more genetically diverse (Additional file 10). No correlation between genetic diversity and distance to origin could be found (R = 0.02, *P* = 0.446) (Additional file 11). This suggests that diversity was not lost as cultivated rye spread from its centre of origin.

### Chloroplast SSRs

Population structure was also studied using cpSSRs markers. Because of the non-Mendelian inheritance of the markers, we analysed the genetic structure by discriminant analysis of principal components (DAPC), a type of analysis that requires no assumptions of Hardy-Weinberg equilibrium and is thus more suited for non-nuclear markers than other methods of assessing population structure.

The DAPC analysis of the cpSSR data could not find a distinct number of clusters with high fit. The DIC value showed some tendencies to plateau at K ≈ 4 and K ≈ 10 for the full dataset. However, the clustering became strongly erratic for K > 3. DAPC clustering did not reflect biological type or taxonomic groups, with the exception of *S. strictum* and *S. africanum* that formed a separate cluster (2). Interestingly, at higher levels of K the Finnmark accessions NGB13868 and NGB14283 stood out from the rest of the *S. cereale*, much like for the SNP markers, and when studying the full set they frequently clustered together with the *S. strictum* accessions rather than other *S. cereale* (Additional file 12). When analysing only *S. cereale* accessions the DIC value had a weak plateau at *K* = 5, which resulted in a structure reminiscent of the nuclear SNPs, albeit less clear (Additional file 13).

### Diversity distribution in areas of contrasting cultivation intensity

To compare areas where rye has been cultivated with high intensity with areas of much more limited rye cultivation, we analysed additional subsets of landrace rye. The Southern subset consisted of seven accessions from Iberia and Morocco and the Northern subset of fifteen accessions from Fennoscandia and western Russia. Among the Southern accessions results indicated discrete populations with each accession belonging to a distinct cluster with little admixture. With the seven accessions *K *= 7 was indeed indicated to be the most likely model (Additional file 5). This homogenous clustering of accessions indicated a degree of reproductive isolation (Fig. 2d). In the case of the Northern accessions *K* = 2 was the best supported model (Additional file 5). As expected, one cluster consisted of the previously identified individuals from Finnmarken (NGB13868 and NGB14283), whereas all other accessions formed a second cluster (data not shown). Removing the highly deviating Finnmarken accessions, *K* = 4 had the highest support (but with strong support also for *K* = 2). At this level of clustering some accessions were grouped solely into a single cluster (though with limited geographic structure), but the individuals in general showed a higher degree of admixture than the Southern accessions at the same level of clustering (Fig. 2e).

**Fig. 6:**
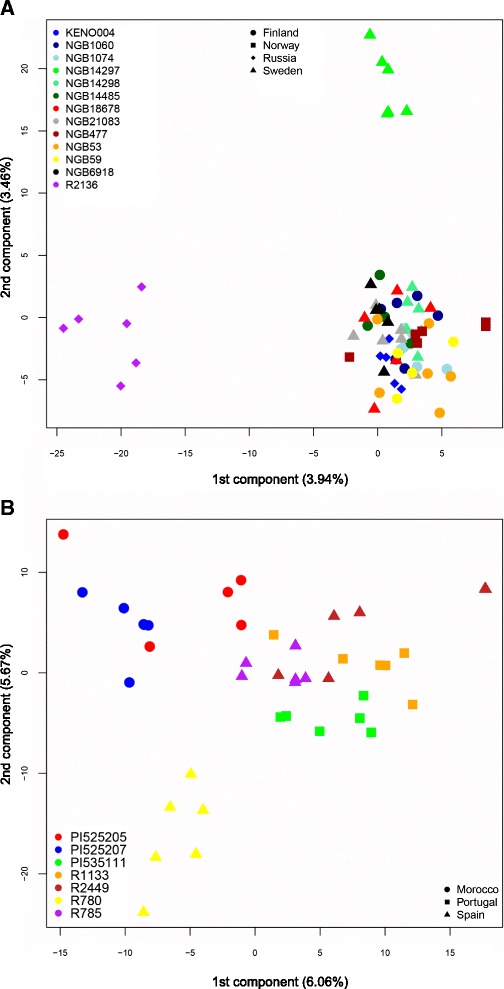
Plot of the 1^st^ and 2^nd^ components of a PCA analysis from contrasting geographic regions. Each point is an individual coloured by accession and shaped according to country of provenance. **a** Fennoscandian (Finland, Norway and Sweden) and Russian landrace accessions, **b**) Iberian (Portugal and Spain) and Moroccan accessions

PCA plots based on genotypes for each separate individual of the accessions illustrate the difference in accession distinction further (Fig. 6). In the Southern dataset individuals from the same country and even accession grouped together to a large extent (Fig. 6a). In the Northern data set two accessions (NGB14297 and R 2136) grouped separately, but the individuals from the remaining accessions, originating from all countries, were mixed (Fig. 6b). Looking at dispersion in PC space, the Norwegian accession NGB477 is much more homogenous than the other accessions (Additional file 1), but excluding this accession, the PC dispersion of individuals from accessions in the Northern group is significantly higher than that of individuals in the Southern group (unpaired *t*-test, *P* < 0.05). This is also reflected by the average within-accession diversity H_E_ that is significantly higher in the Northern group than in the Southern group (unpaired *t*-test, *P* < 0.01). Additionally, F_ST_ values between accessions in the Northern group (average 0.067) were significantly lower than values between accessions in the Southern group (average 0.090) (unpaired *t*-test, *P* < < 0.001).

In summary, accessions from the Southern group are genetically differentiated from each other and with somewhat lower within-accession diversity. The Northern group consists both of some accessions clearly differentiated from others but with the majority of accessions belonging to virtually the same population.

### The effect of pooling on the detection of population structure

The structure detected when analysing the *in silico* pooled data in general showed good agreement with the structure detected when analysing the separate individuals dataset. However, the level of detail at which structure could be detected was lower for the pooled data set (Additional file 14). *∆K* and CLUMPP H' values typically supported lower levels of clustering for pooled than un-pooled data (Additional file 5). A notable exception was the Northern accessions where a high number of clusters were supported (8 including the Finnmarken accessions and 7 excluding them). However, level of clustering above *K* = 2 added no biologically relevant information and only resulted in split ancestry of accessions. In the southern group no population structure could be detected for the pooled data set (Additional file 14). One consequence of the *in silico* pooling thus seems to be a reduced power to detect geographic structuring.

## Discussion

### *Genetic diversity in the genus* Secale

In this study we demonstrate that SNP markers developed for rye elite cultivars can be applied to landrace varieties as well as feral and wild ryes. In contrast to many previous investigations of rye, using single individuals or pooling schemes, we also assess within-accession genetic diversity and structure in rye populations. One of the most striking results is the wide distribution of the diversity found within accessions of rye. AMOVA showed that the absolute majority of the genetic diversity was present within accessions with only little additional diversity present within taxon, types or geographic regions. From a genetic diversity perspective conservation of landrace rye from a reasonably limited regional area might be as efficient as conserving additional taxon or landraces from a wide geographic range, at least when Northern accessions are concerned. The cross-pollinating reproductive habit of rye most likely plays a contributing role in this.

Our results regarding genetic diversity in different taxa must, however, be interpreted with some caution. In our panel, the genetic diversity is higher in cultivated rye than in feral, and wild *S. strictum* accessions have the lowest diversity (Table 2). This is contrary to the expectation of higher genetic diversity in wild or feral accessions, unaffected by a domestication bottleneck and observed in many other species [e.g. 51–53]. This unexpected difference could be due to ascertainment bias as the SNP panel was originally developed for elite cultivars of cultivated rye. Strong effects of ascertainment bias when comparing materials with different improvement status have been noted previously in both barley and wheat [31, 54, 55].

We do consider ascertainment bias in our material to be present, but not affecting different taxa very differently. First of all, the higher gene flow of outcrossing crops such as rye lower the effects of ascertainment bias as genetic diversity is less structured and more evenly distributed within and between taxa (Fig. 2). Second we note that almost all markers detect diversity and that the proportions of monomorphic markers are rather low in all taxa with the exception of *S. africanum* and *S. strictum*. Third the minor allele frequency distribution was similar in different status groups (Additional file 4) indicating that the ascertainment bias had comparable effects on the different status groups. Additionally, merging SNPs into haplotypes has been shown to alleviate the effects of ascertainment bias when present [49]. Our merging of SNPs to haplotypes had, for most taxa, little effect on the relative differences in diversity. Only *S. africanum* and *S. strictum* showed a relative increase in diversity when SNPs were merged into haplotypes of increasing length (Fig. 1). Based on this we believe that ascertainment bias is primarily a problem in the cases of *S. africanum* and *S. strictum*. A higher diversity in cultivated rye than in feral types has also been found using less biased markers such as AFLPs [14], SSRs [15] and ISSRs [56] . This suggests that the limited diversity detected in at least feral ryes is not primarily due to ascertainment bias but that the feral genetic diversity might rather be poorly represented in genebank collections.

The within-accession diversity measures largely mirror those for total diversity, with lower H_E_ in the wild and feral taxa. The exception is the wild *S. vavilovii*, with average within-accession H_E_ in same range as cultivated *S. cereale* and significantly higher than the feral taxa. Among the cultivated accessions, most accessions have similar levels of diversity and no significant differences were found between the different agro-climatic regions. Somewhat surprising, H_E_ was not lower in the commercial cultivars than in the landraces. Similar observations were, however, made by Persson & von Bothmer [22] using isozyme markers. This is contrary to inbreeding crops, where landraces regularly have much higher within-accession diversity than commercial breeds [57]. The accession NGB13868 has a markedly low H_E_. This accession is known to originate from only a few seeds, which explains its reduced diversity [58].

As an alternative measure of within-accession diversity we studied PC dispersion. Pairs of individuals which are genetically similar will be geometrically close within PC space. Comparing PC dispersions between accessions gives a measure of the difference in relative diversity. The variance of the accession’s pair-wise distances further reveals if the distances giving rise to the PC dispersion are randomly distributed. PC dispersion means are mostly correlated to H_E,_ but not necessarily so. For example, the accession NGB477, has high H_E_ but deflated PC dispersion means. This means that although the accession contains a lot of diversity, all individuals are relatively similar to each other. Some accessions have inflated PC dispersion variance, caused by two or more individuals in the accession being highly similar. If two individuals have increased similarity an origin as siblings from the same ear could be expected, not an unlikely situation with low population sizes maintained in genebank. Very highly similar or identical individuals, however, could be considered clones originating from the same embryo. One explanation could be presence of polyembryonic seeds [59], giving rise to several shoots, accidently sampled as different individuals. Re-analysing our data, with the highly similar individuals removed, showed that their effect on genetic diversity and structuring was all non-significant.

### *Insights into* Secale *taxonomy*

The taxonomic classification within the genus *Secale* was long elusive. However, most recent molecular studies agree of a three-species model with *S. sylvestre, S. strictum* and *S. secale* [15, 16, 56, but contrasting 12). This taxonomic classification is also in agreement with Frederiksen and Petersen’s [10] morphology-based taxonomy. Our data support this classification, although *S. sylvestre* was not included in the study. We furthermore find support for the sometimes suggested species *S. africanum* and *S. vavilovii* (see e.g. [11] and references therein) as subspecies of *S. strictum* and *S. secale* respectively. In this study, the *S. africanum* accession clustered both in STRUCTURE and with PCA together with the true *S. strictum* accessions. The relatively low genetic distance (0.051) and F_ST_ value (0.133) between *S. africanum* and *S. strictum,* smaller than to any other taxa, reinforced the close relationship. Although found only in South Africa *S. africanum* is inter-fertile with the remaining *Secale* taxa [60]*.* The proximity between *S. africanum* and *S. strictum* accessions strengthen the hypothesis that the odd location of *S. africanum,* far away from the distribution of other *Secale* taxa, is better explained by human activities, namely the introduction of rye and its weeds in South Africa by European settlers, rather than it being a remnant of an originally much larger distribution area of *Secale* [10].

*S. vavilovii* plants are fully shattering, with ears that disarticulate spontaneously, and can be considered a wild subspecies and not just a weedy or feral type [17]. Nonetheless, *S. vavilovii* clusters closely with cultivated *S. cereale* accessions both in the STRUCTURE analysis and PCA (Fig. 2a, Fig. 4, Additional file 6) and must be considered to be a subspecies of *S. cereale.* The low F_ST_ and genetic distance between *S. cereale and S. vavilovii* (0.016 and 0.017 respectively) attest to their relatedness and low genetic differentiation (Table 4). Cultivated rye has been proposed to derive from either *S. strictum* (Persl.) [= *S. montanum* Guss.] or from *S. vavilovii* in Eastern Turkey and Armenia [17, 61]. Our results confirm previous studies [11, 15] suggesting that *S. vavilovii* is the most likely wild ancestor of cultivated rye.

The classification of *S. secale* subspecies has been more inconclusive, particularly the relationship of feral and cultivated rye forms [7–9]. Our analyses support grouping the cultivated and the feral ryes as the same species and that all these taxa cross-hybridise to some degree. Although feral rye present a certain degree of genetic distinctiveness from both cultivated and wild ryes, in congruence with Chikmawati et al. [14], we conclude that geographic origin is more relevant for population structure than taxonomic subspecies classification. In our dataset some feral ryes are more similar to cultivated rye from the same region than landraces are to other landraces from different regions. For example, the *S. afghanicum* accession R 777 has a very low F_ST_ (0.096) from the Afghan landrace PI 220119; the Spanish *S. segetale* R 779 has one of its lowest pairwise F_ST_ (0.087) with the Spanish landrace R 785 (Additional file 7), a value lower than those comparing R 779 with other *S. segetale* accessions. The potentially mislabelled accession of *S. strictum* (PI 240285) mentioned above is described in the passport data as *S. strictum* subsp. *anatolicum*), from Turkey and clusters together with feral and cultivated rye from Turkey, Afghanistan and Pakistan and one cultivated rye from the same region (turquoise in Fig. 2a). A similar pattern is painted in the PCA where, although some feral accessions were genetically distinct enough to constitute their own clusters, most feral accessions clustered with the cultivated landraces from the same regions (for example *S. segetale* from Spain, *S. ancestrale* from Turkey or *S. afghanicum* from Afghanistan) rather than with accessions of the same taxon.

Feral rye phenotypes have been described to morphologically diverge quickly from cultivated rye progenitors, especially if cessation of rye cultivation in one region reproductively isolates feral types from and cultivated ones [62]. In this study we can se how feral ryes share some alleles with both *S. vavilovii* and *S. cereale* landrace accessions. These results suggest a complex scenario of high introgression between the ryes in the *S. cereale – vavilovii* group, but with no detectable contribution from the *S. strictum – S. africanum* group.

### Geographic structuring of genetic diversity in cultivated rye

Previous studies of the distribution of genetic diversity in cultivated rye have mainly failed to find any clear geographic patterns [13, 14, 22, 23, 63]. Bolibok-Bragoszewska et al. [24] found separation between Near East rye and European rye but no geographical clustering within Europe. The very low degree of variation distributed between regions, shown by AMOVA, suggests that a large number of markers are needed to identify geographical structure. The SNP panel used here, in combination with the genotyping of multiple individuals from each accession, does, however, seem sufficient to cluster European landraces geographically (Figs. 2c and 3). It is also clear that although 80 % or more of the genetic diversity present can be accounted for in pooled samples, the power to detect geographic structure is reduced by pooling of samples before genotyping. We were consistently able to detect higher levels of clustering using our un-pooled data set than we were with our *in silico* pooled data. This supports the results of computer simulations [64].

The five major groups of landrace rye detected, partly reflects the agro-climatic zones of Europe (as e.g. proposed by Bouma [41]). However, a distinct cluster of accessions ranging from Scotland in the North to Morocco in South along the Atlantic coast represents areas with rather different climates (blue in Fig. 3). Likewise, a cluster of accessions from Scandinavia, Finland and Russia also span across agro-climatic zones (yellow in Fig. 3). Thus, the geographic distribution of genetic diversity is not only formed by agro-climatic suitability, but also through pollen dispersal and seed exchange in areas with closer cultural contacts.

The smallest cluster, consisting of a single Norwegian and a single Swedish landrace, both from the Finnmarken area stood out as the geographically most local group. Most of the individuals in these accessions also shared the same, and amongst cultivated rye unique, chloroplast genotype. The distinctiveness of landrace rye from this area from the majority of Scandinavian rye was previously noted by Persson et al. [23]. The rye cultivated in this region is strongly connected to historical migrations of people. This region of Norway and Sweden is known as the Finnmarken area, the area where Finnish farmers settled after leaving their native country in the 16^th^ century. It is well known that the Finnish immigrants practiced slash-and-burn cultivation of rye in this area and also brought seed with them from their native area [6]. The accession NGB13868 also has the local name “Finn-rug”, meaning Finnish rye. Rye aimed for slash-and-burn cultivation is often characterized by extreme tillering capacity, a trait also observed in the “Finn-rug” accession [58]. We were, however, unable to detect any significant clustering between the Finnmarken rye and any of the Finnish accessions included in this study. It should be noted, however, that our study also includes other Scandinavian rye landraces with passport data describing them as slash-and-burn types [23]. These accessions do not cluster genetically with the accessions from Finnmarken, but instead with other Nordic accessions aimed for cultivation on ordinary cropland. One hypothesis is that the rye accessions from the isolated region of Finnmarken represent an older type of slash-and-burn rye, whereas the other slash-and-burn rye landraces in Fennoscandia have been outcrossed and mixed with more abundant rye types.

The population structure detected suggests that European rye landraces could have originated in different places. According to the *K* = 9 STUCTURE model (Fig. 2), the yellow cluster that includes most Scandinavian and Russian accessions also contains a part of the diversity of some *S. vavilovii* individuals from Afghanistan as well as feral *S. segetale* and *S. afghanicum* from the same country. In a study screening RAPDs in a panel of worldwide breed and landrace ryes, Ma et al., [63] found that northern European rye accessions (Scandinavia and Germany) clustered together and in this cluster a landrace from Afghanistan was also included. It is possible that the rye introduced to Russia and Northern Europe originated in the area around Afghanistan and was introduced through a route north of the Caspian and Black seas, whereas rye in the south of Europe originated in the Turkish area. Alternatively, rye could have originated in one core area in the Near East and, after spreading to Europe, gone through bottlenecks and distinct selection pressures leading to an adaptation to different regions. Such population dynamics could also generate the distinct clusters observed in cultivated rye. A strong correlation between genetic structuring and agro-climatic regions would then be expected. We observe increasing genetic diversity within landraces from more northern latitudes (Additional file 10). In spite of necessary adaption to a climate much different from the climate at the center of origin, rye cultivated in the North contain as much diversity as rye cultivated close to the center of origin. Possibly the more intense rye cultivation in the North until recent time has contributed to maintain diversity in landraces.

A distinguishing feature during adaption of rye in Europe would have been selection for winter versus spring-sown ryes, with the South and West of Europe ryes being predominantly spring (or facultative) types and northern European ryes being winter types. Ma et al. [63] found that the winter vs spring habit generated two very distinct clades in their panel of 42 elite breeds. Contrastingly, in their analysis of Swedish historical and genebank kept landraces, Hagenblad et al. [65] did not observe any separation between winter and spring types of rye. In this study, PCA of cultivated rye separated spring from winter varieties along the 1^st^ PC although some overlap was observed (Additional file 15). It is possible that in different regions of Europe the choice of cultivating spring or winter rye reflected local climatic specificities and agronomic practices leading to a slight genetic differentiation over time. We note also that STUCTURE groups in Central and South Eastern Europe contain both spring and winter forms.

The STRUCTURE assignment of the three modern varieties reflected their breeding history. NGB 6431 ‘Kungs II’ clustered among landraces from Northern Europe. This variety was breed in Sweden from landraces from Northern Germany, possibly pollinated with Swedish landraces. PI428373 ‘Petkus’ spring rye, clustered among other spring rye landraces from Western Europe. PI 323382 ‘Imperial’ clustered among landraces from the Central Europe. ‘Imperial’ is an American variety selected from the landrace ‘Schlanstedt’, originating in central Germany.

Mitochondrial and chloroplast markers have been used to contrast maternal spread (in plants through seeds) with biparental spread (through both seed and pollen). In rye, exclusively maternal transmission of chloroplasts has been detected in some studies [66, 67]; while others have reported biparental inheritance[68–70]). In rye the structure detected using cpSSR should thus probably be considered to reflect both seed spread and to some extent pollen movement. Studies combining nuclear and chloroplast markers sometimes provide similar information about a species’ evolutionary narrative, but can potentially provide scenarios that are contrasting, for example in rice [71], *Carex* [72], almond [73] and *Arabidopsis* [74]. We carefully analysed all electropherograms for the cpSSRs. However, some alleles detected differed by only a single base-pair and errors resulting from PCR amplification cannot be ruled out. The cpSSR data, based on only a few markers, and DAPC clustering must therefore be considered with caution. The structure could, however, lend additional support to the structure as determined by the SNP markers. Except for *S. strictum* and *africanum*, we observed no taxonomic clustering, again supporting the inclusion of all feral taxa, *S. vavilovii* and cultivated rye in the same species. Among the cultivated landraces, accessions from Western Europe, Northern Europe and South Eastern Europe formed three groups, suggesting seed exchange within these areas and supporting nuclear SNP geographic patterns. Among the accessions from Finnmarken all individuals of NGB13868 and half of the individuals in NGB14283, had a distinct cp-genotype not found elsewhere among cultivated rye. This suggests a foreign, but unknown, origin for these landraces. Screening additional landraces, especially from remote areas in Finland, with the cpSSR markers, or the full set of SNP markers, could shed light on the origin of these ryes and the possible connection to Finnish migration in the 16^th^ century.

### Contrasting genetic structuring in regions reflects past cultivation intensity

Our results demonstrate how cultivation practices can affect how genetic diversity is distributed in regions. Geographic structuring was investigated at a regional level using two contrasting groups of accessions. The Southern group consisted of spring type rye from Iberia and Morocco, all belonging to the same Structure group (blue in Fig. 3). The Northern group consisted of winter type rye from Fennoscandia and Western Russia, belonging to the yellow Structure group. We concluded from PCA, F_ST_ and PC dispersion analysis that accessions in the Southern group were much more genetically distinct from each other than the majority of accessions in the Northern group (Fig. 6; Additional file 1 and Additional file 3).

A distinct clustering of landraces suggests more limited gene flow between accessions. In Iberia and Morocco rye is only cultivated as a minor crop in mountainous regions [75, 76]. Contrastingly, in Fennoscandia rye was the dominant food crop from the 16^th^ century until the mid-20^th^ century [5], which would allow more frequent gene flow between populations. From our data we cannot say if the higher gene flow in Northern group is due to more seed exchange, more pollen dispersal or both. Unfortunately, the power from the limited number of maternally inherited markers is not enough to detect different patterns of pollen and seed dispersal within this region. From studies of landrace dynamics in inbreeding crops, such as pea [57] and rice [77] we do, however, know that isolation by distance or decreased cultivation intensity can rapidly cause genetic drift and differentiation between populations. These processes are probably slower in cross-pollinating crops, but the same trend that can be seen in the Southern group and could be expected in other areas where rye cultivation declines.

## Availabiliy of supporting data

The SNP and chloroplast SSR genotyping data generated for this paper are available at the Dryad data archive (https://datadryad.org/resource/doi:10.5061/dryad.tn2fb).

## Additional files

Additional file 1:
**Accessions used in this study, genebank provider, taxon, provenance and growth habit and genetic diversity measures.** (XLSX 29 kb) Additional file 2:
**List of SNPs genotyped.** Assembly, contig and position in contig is indicated in SNP names. The SNP within brackets and flanking sequences are shown. (XLSX 65 kb) Additional file 3:
**Minor allele frequency distributions for A)** ***S. cereale*** **, B)** ***S. strictum*** **, C)** ***S. vavilovii*** **, D)** ***S. africanum*** **, E)** ***S. dighoricum*** **, F)** ***S. ancestrale*** **, G)** ***S. afghanicum*** **and H)** ***S. segetale*** **respectively.** Bars show the observed number of markers with a given minor allele frequency. (PDF 51 kb) Additional file 4:
**Minor allele frequency distributions for A) Wild rye, B) Feral rye and C) Cultivated rye respectively.** Bars show the observed number of markers with a given minor allele frequency and the red line depicts the distribution expected under neutrality. (PDF 6 kb) Additional file 5:
**Determination of the best-fit STRUCTURE models by determination of** ***ΔK*** **values [** 46 **] and H' values obtained with CLUMPP [** 45 **] for data analysed as separate individuals and individuals pooled** ***in silico. ***(PDF 969 kb) Additional file 6:
**STRUCTURE output for** ***K*** **models 2 to 12 for the complete set of accessions.** Accessions are organized by taxon. (PDF 11269 kb) Additional file 7:
**Pairwise F**_**ST**_ **values between pairs of accessions.** (XLSX 45 kb) Additional file 8:
**Correlation between geographic distance (km) and genetic distance (D**_**S**_ **) [** 77 **] between individual rye accessions.** (PDF 1415 kb) Additional file 9:
**Correlation between longitude (°) and genetic diversity (H**_**E**_ **) in rye accessions.** (PDF 598 kb) Additional file 10:
**Correlation between latitude (°) and genetic diversity (H**_**E**_ **) in rye accessions.** (PDF 622 kb) Additional file 11:
**Correlation between distance to origin of domestication (km) and genetic diversity (H**_**E**_ **) in rye accessions.** (PDF 670 kb) Additional file 12:
**DAPC output based on chloroplast SSRs for** ***K*** **models 2 to 6 for the complete set of accessions.** Each bar is one accession averaged on 4 to 6 individuals. Accessions are organized by taxon. A) *K* = 2, B) *K* = 3, C) *K* = 4, D *K* = 5, E) *K* = 6 (PDF 11334 kb) Additional file 13:
**Geographical distribution of cultivated rye landrace clusters according to the DAPC** ***K*** **= 5 model.** Each landrace is depicted as a pie chart with the proportional membership of its alleles to each one of the five clusters. (JPG 1027 kb) Additional file 14:
**Clustering of rye accessions based on STRUCTURE analysis of** ***in silico*** **pooled data for** ***K*** **values corresponding to those shown in Fig.** 2 **.** A) *K* = 9 model for the complete set of accesions. B) *K* = 7 model for the Southern set of Moroccan, Portuguese and Spanish landraces. C) *K* = 4 model for the Northern set of Fennoscandian and Russian landraces. (PDF 61 kb) Additional file 15:
**PCA of cultivated rye accessions coloured by their growth habit (winter vs spring).** (PDF 1436 kb)
