# Supplementary material for: Geographical distribution of genetic diversity in Secale landrace and wild accessions
Source: BMC Plant Biol. 2016 Jan 19;16:23. doi: 10.1186/s12870-016-0710-y (PMC4719562; doi:10.1186/s12870-016-0710-y)
Supplement: Additional file 3: — Minor allele frequency distributions for A) S. cereale , B) S. strictum , C) S. vavilovii , D) S. africanum , E) S. dighoricum , F) S. ancestrale , G) S. afghanicum and H) S. segetale respectively. Bars show the observed number of markers with a given minor allele frequency. (PDF 51 kb) [file 12870_2016_710_MOESM3_ESM.pdf]

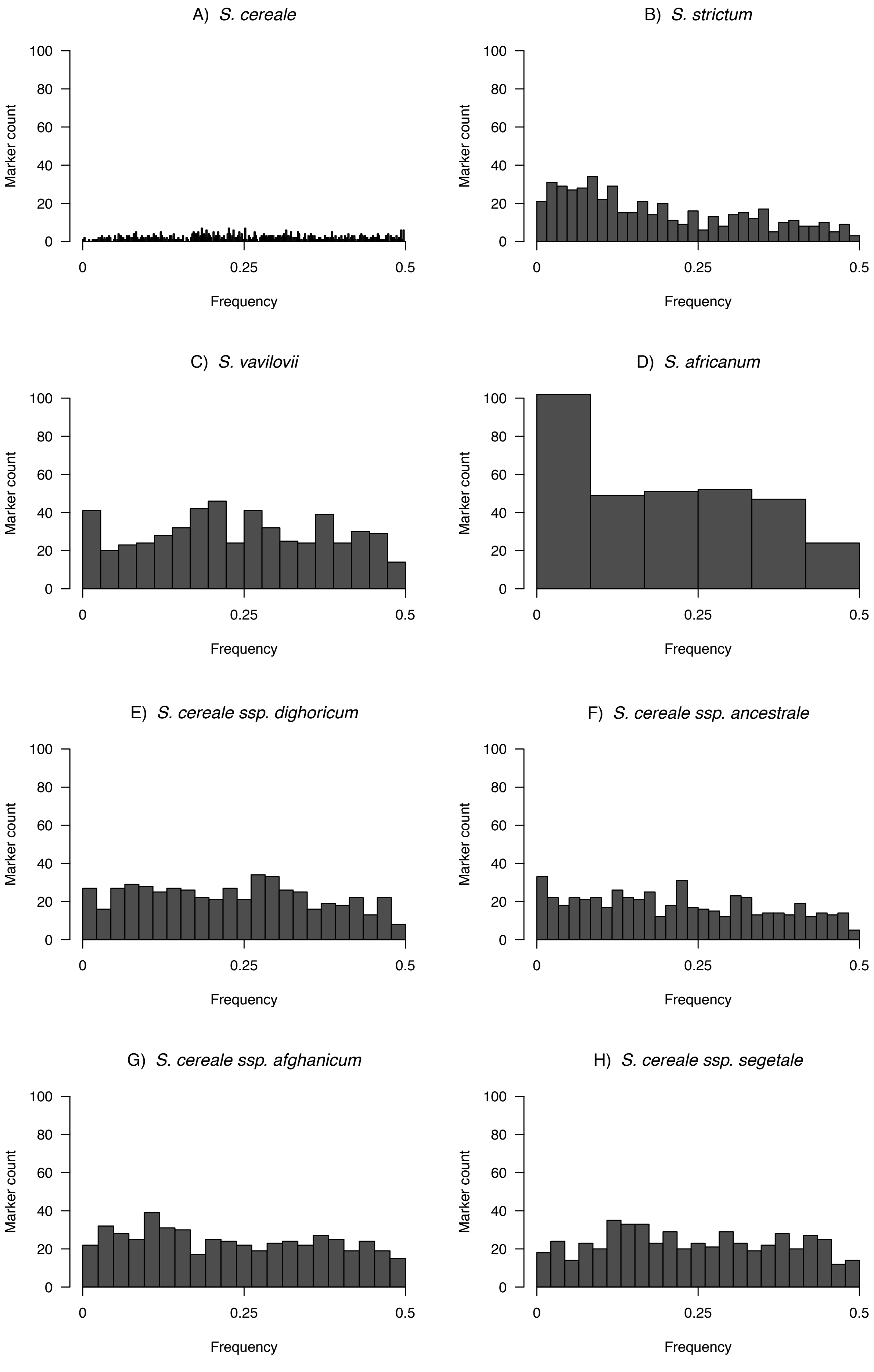

A) S. cereale
B) S. strictum
C) S. vavilovii
D) S. africanum
E) S. cereale ssp. dighoricum
F) S. cereale ssp. ancestrale
G) S. cereale ssp. afghanicum
H) S. cereale ssp. segetale
Marker count
Frequency
0
20
40
60
80
100
0.25
0.5
