# Supplementary figures and images for: Geographical distribution of genetic diversity in Secale landrace and wild accessions
Source: BMC Plant Biol. 2016 Jan 19;16:23. doi: 10.1186/s12870-016-0710-y (PMC4719562; doi:10.1186/s12870-016-0710-y)

### A) Wild rye

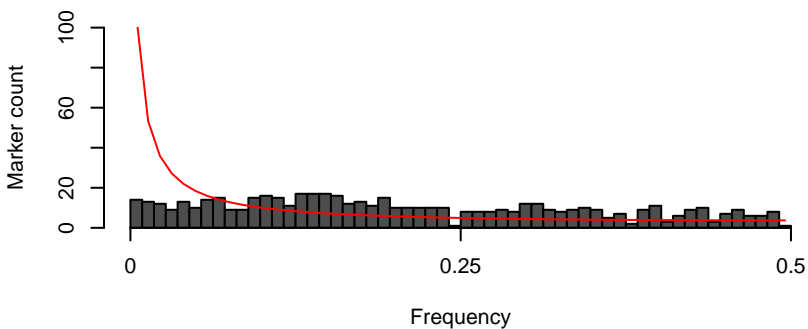

### B) Feral rye

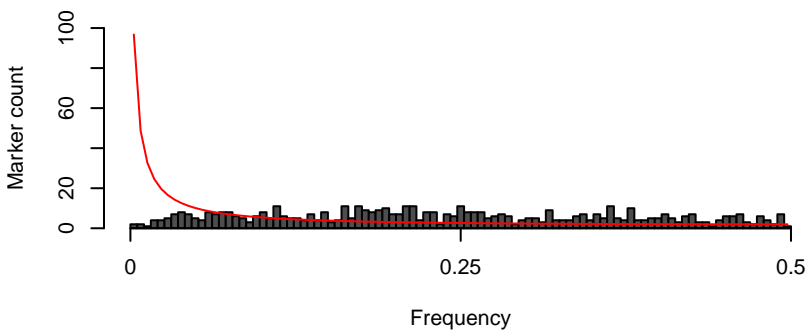

### C) Cultivated rye

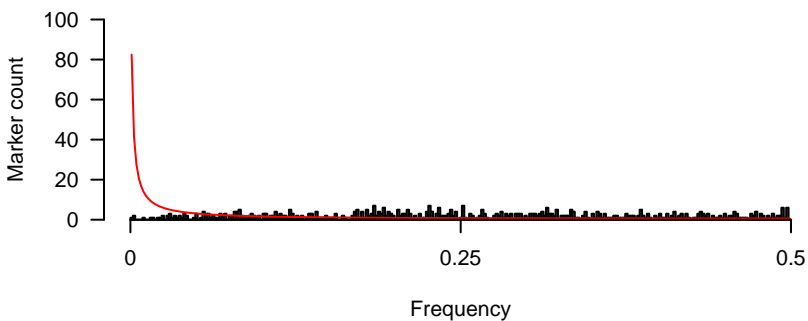

Supplement: Additional file 4: — Minor allele frequency distributions for A) Wild rye, B) Feral rye and C) Cultivated rye respectively. Bars show the observed number of markers with a given minor allele frequency and the red line depicts the distribution expected under neutrality. (PDF 6 kb) [file 12870_2016_710_MOESM4_ESM.pdf]

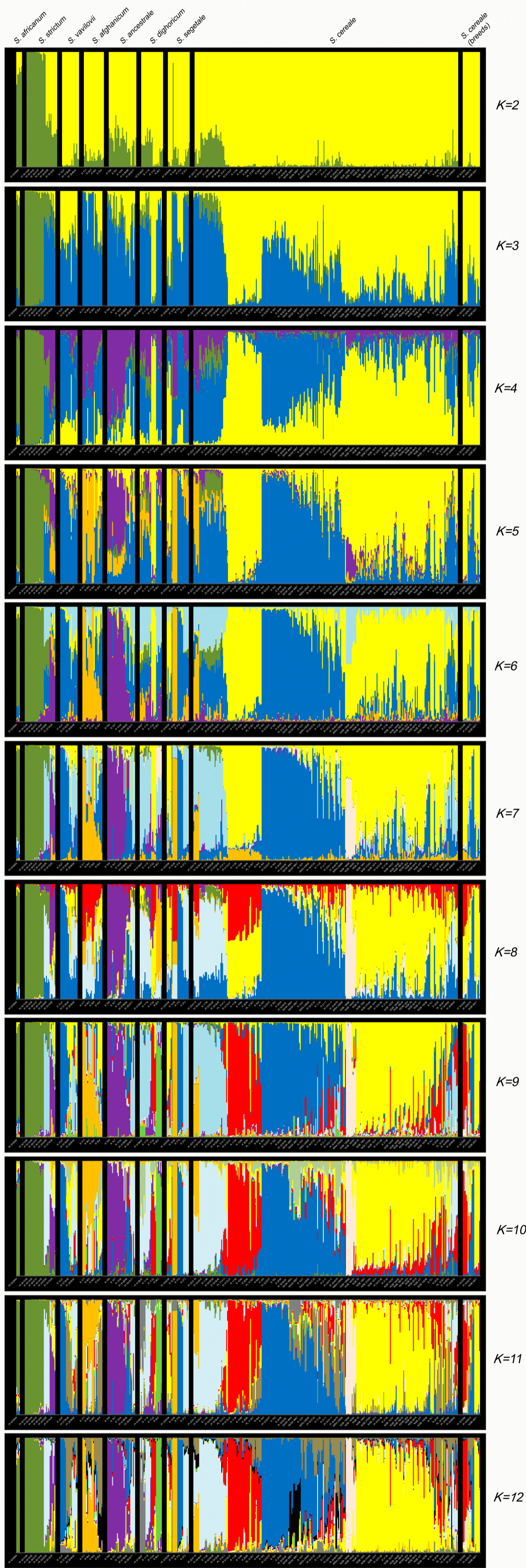

Supplement: Additional file 6: — STRUCTURE output for K models 2 to 12 for the complete set of accessions. Accessions are organized by taxon. (PDF 11269 kb) [file 12870_2016_710_MOESM6_ESM.pdf]

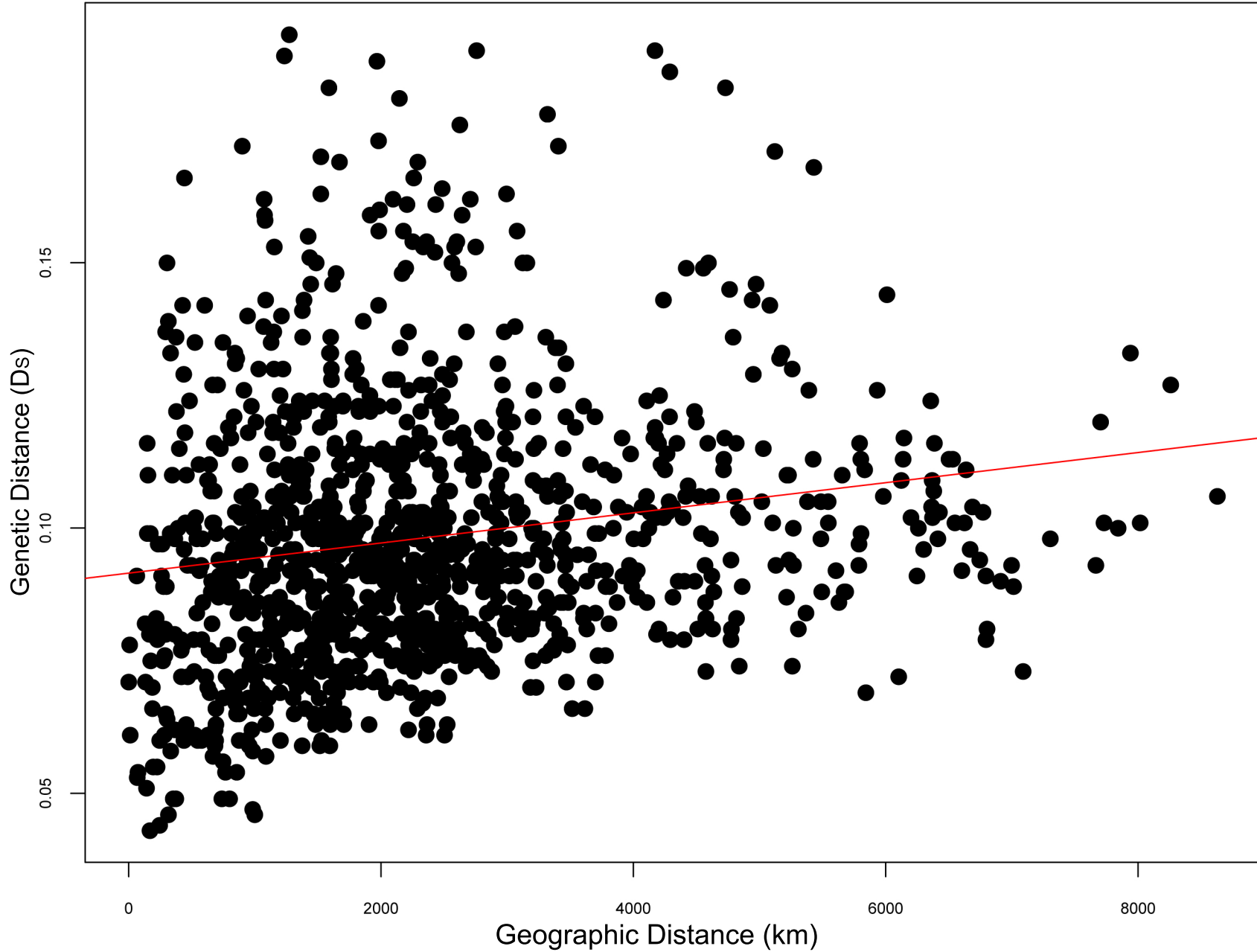

Supplement: Additional file 8: — Correlation between geographic distance (km) and genetic distance (D S ) [ 77 ] between individual rye accessions. (PDF 1415 kb) [file 12870_2016_710_MOESM8_ESM.pdf]

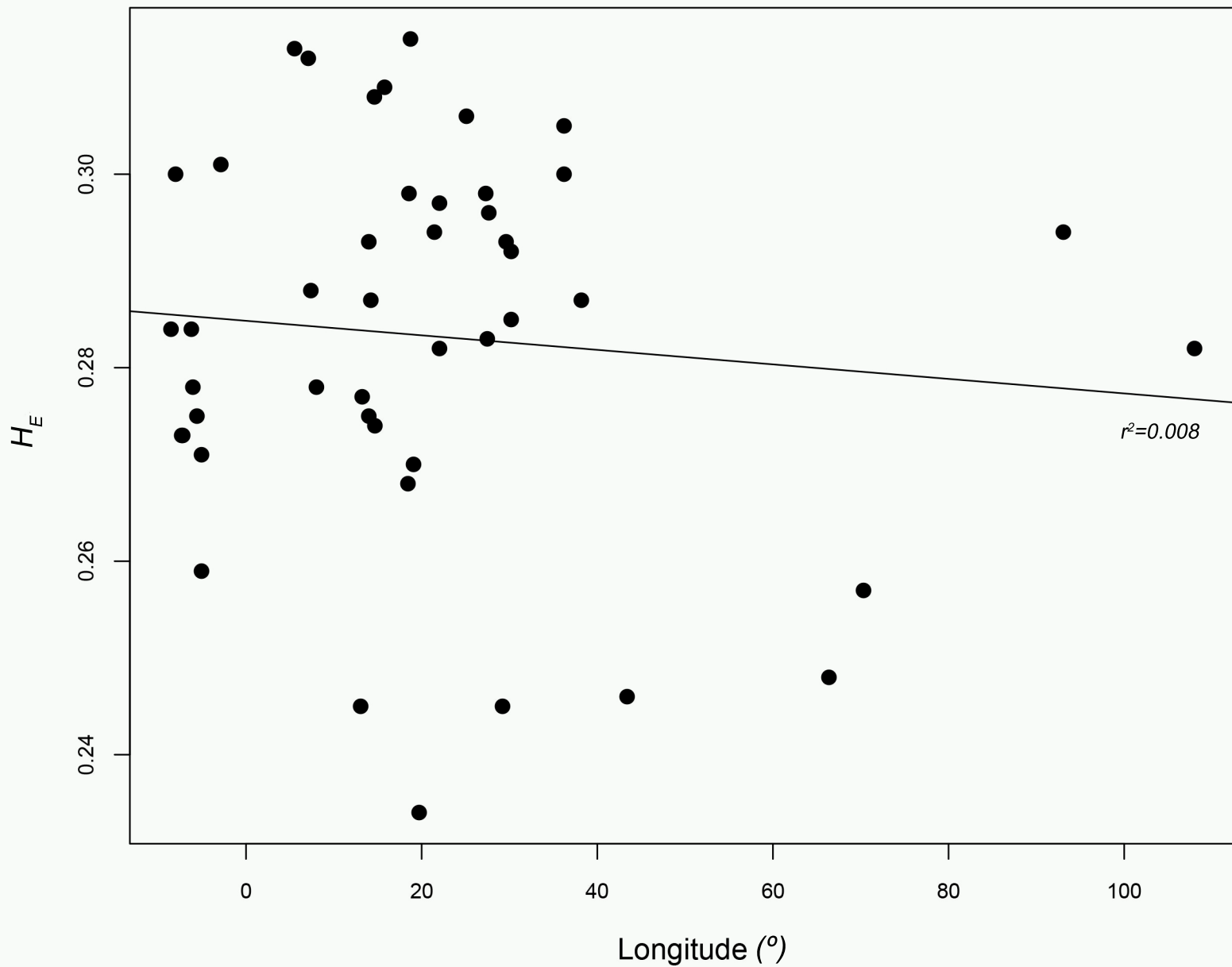

Supplement: Additional file 9: — Correlation between longitude (°) and genetic diversity (H E ) in rye accessions. (PDF 598 kb) [file 12870_2016_710_MOESM9_ESM.pdf]

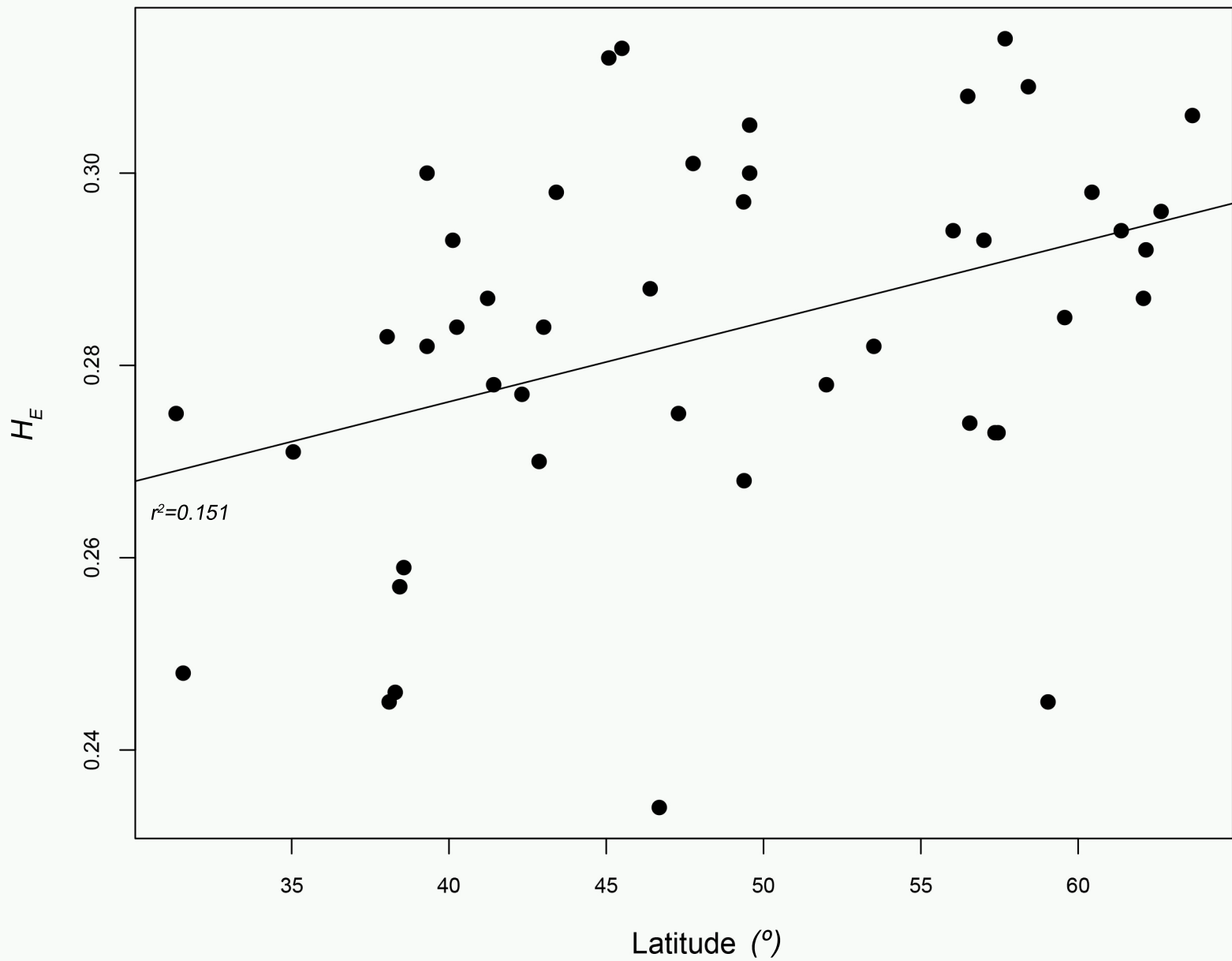

Supplement: Additional file 10: — Correlation between latitude (°) and genetic diversity (H E ) in rye accessions. (PDF 622 kb) [file 12870_2016_710_MOESM10_ESM.pdf]

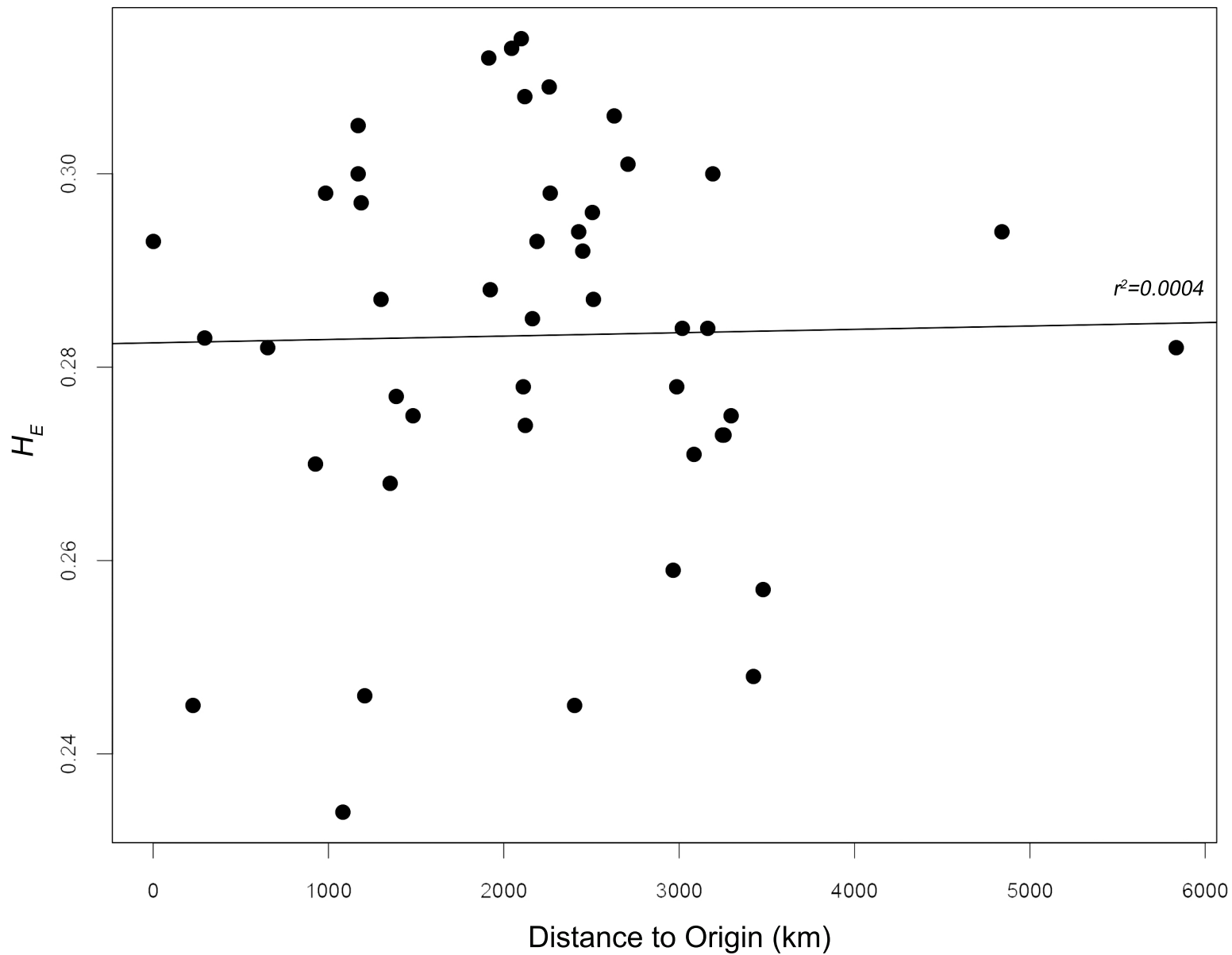

Supplement: Additional file 11: — Correlation between distance to origin of domestication (km) and genetic diversity (H E ) in rye accessions. (PDF 670 kb) [file 12870_2016_710_MOESM11_ESM.pdf]

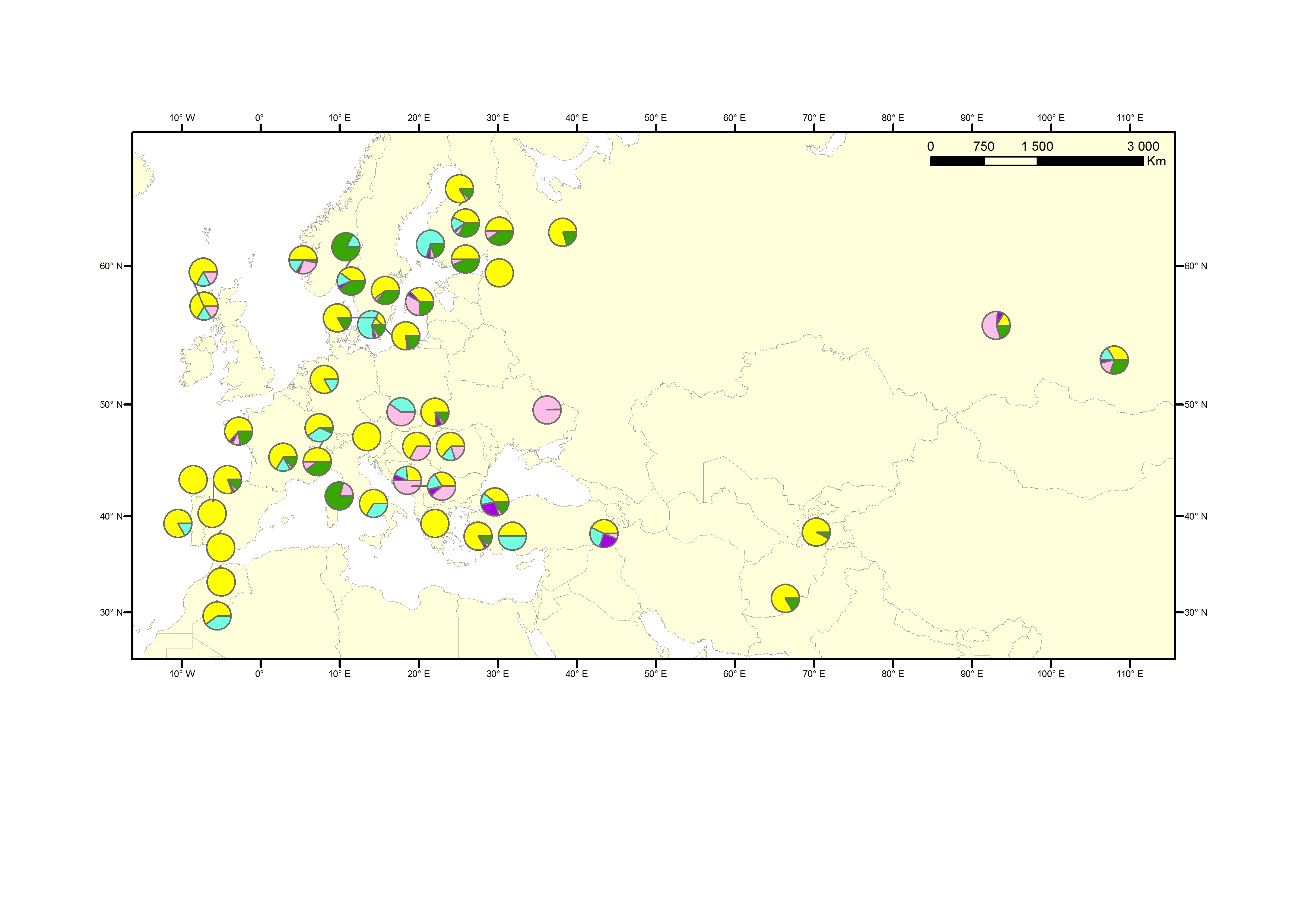

Supplement: Additional file 13: — Geographical distribution of cultivated rye landrace clusters according to the DAPC K = 5 model. Each landrace is depicted as a pie chart with the proportional membership of its alleles to each one of the five clusters. (JPG 1027 kb) [file 12870_2016_710_MOESM13_ESM.jpg]

A

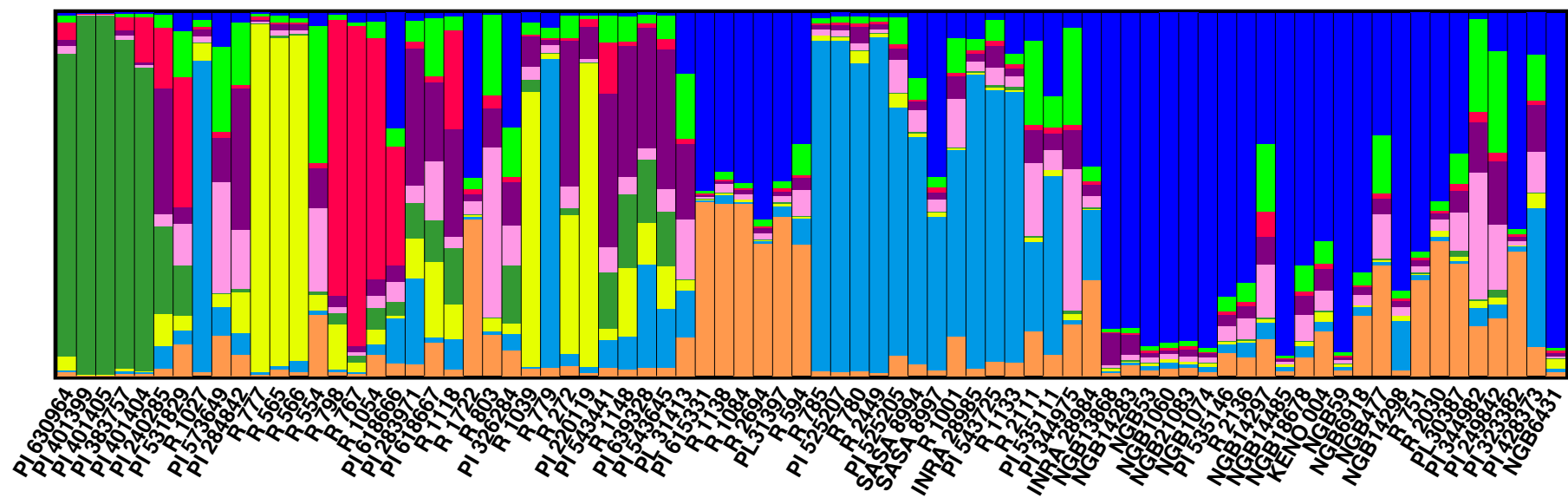

B

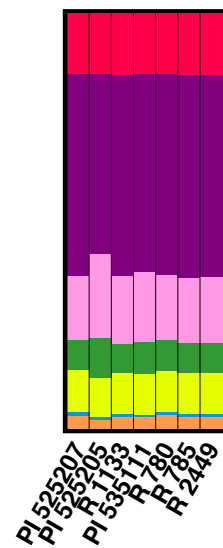

C

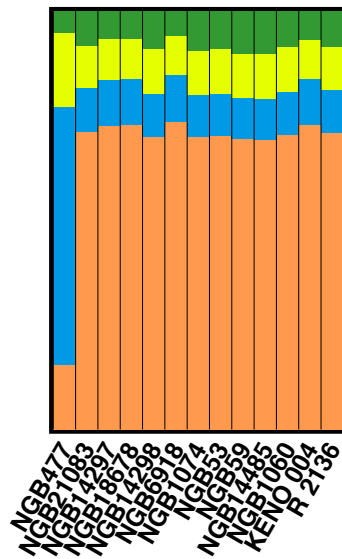

Supplement: Additional file 14: — Clustering of rye accessions based on STRUCTURE analysis of in silico pooled data for K values corresponding to those shown in Fig. 2 . A) K = 9 model for the complete set of accesions. B) K = 7 model for the Southern set of Moroccan, Portuguese and Spanish landraces. C) K = 4 model for the Northern set of Fennoscandian and Russian landraces. (PDF 61 kb) [file 12870_2016_710_MOESM14_ESM.pdf]

2nd component (6.03%)

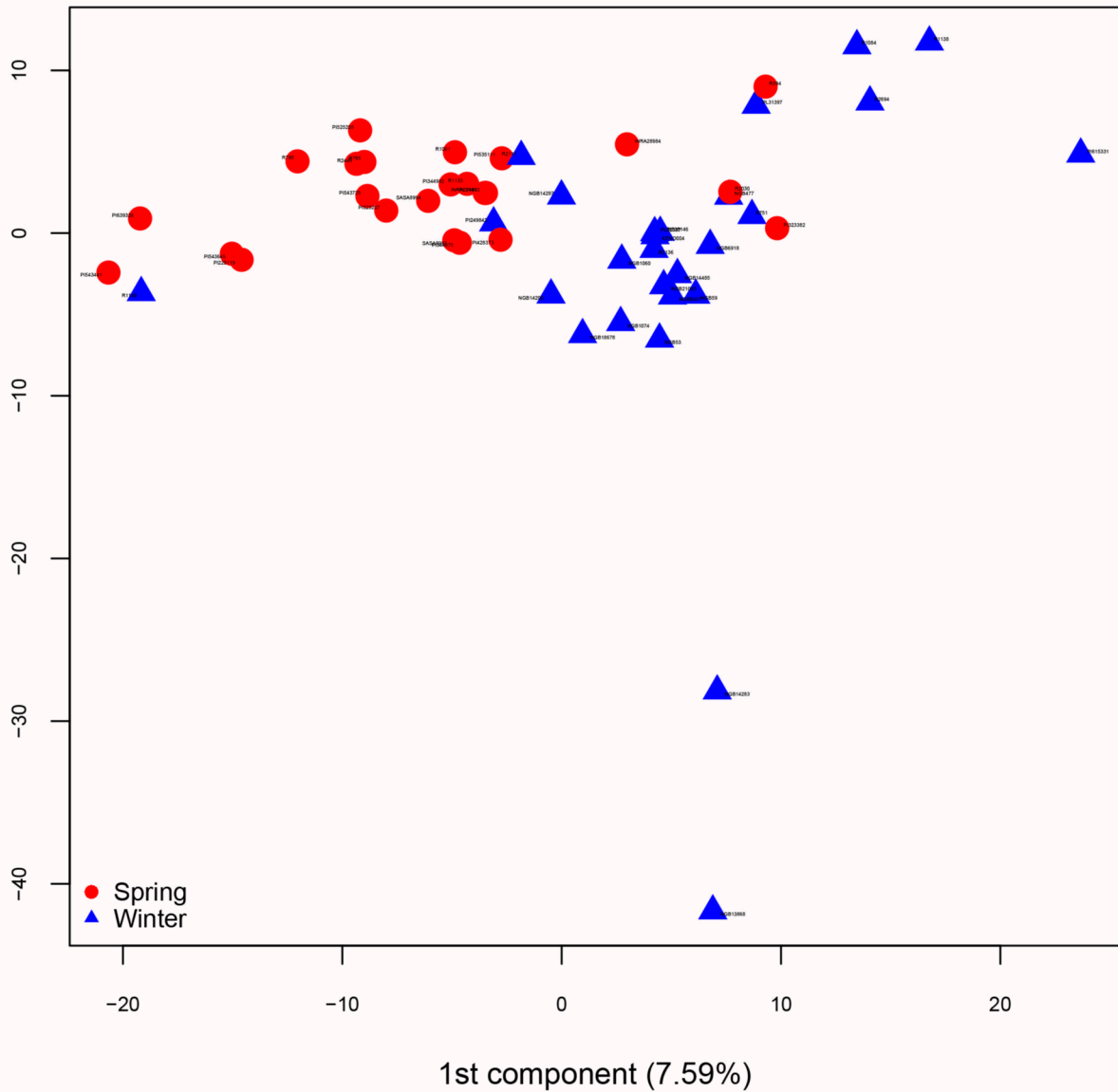

Supplement: Additional file 15: — PCA of cultivated rye accessions coloured by their growth habit (winter vs spring). (PDF 1436 kb) [file 12870_2016_710_MOESM15_ESM.pdf]
