# Supplementary material for: Geographical distribution of genetic diversity in Secale landrace and wild accessions
Source: BMC Plant Biol. 2016 Jan 19;16:23. doi: 10.1186/s12870-016-0710-y (PMC4719562; doi:10.1186/s12870-016-0710-y)
Supplement: Additional file 5: — Determination of the best-fit STRUCTURE models by determination of ΔK values [ 46 ] and H' values obtained with CLUMPP [ 45 ] for data analysed as separate individuals and individuals pooled in silico. (PDF 969 kb) [file 12870_2016_710_MOESM5_ESM.pdf]

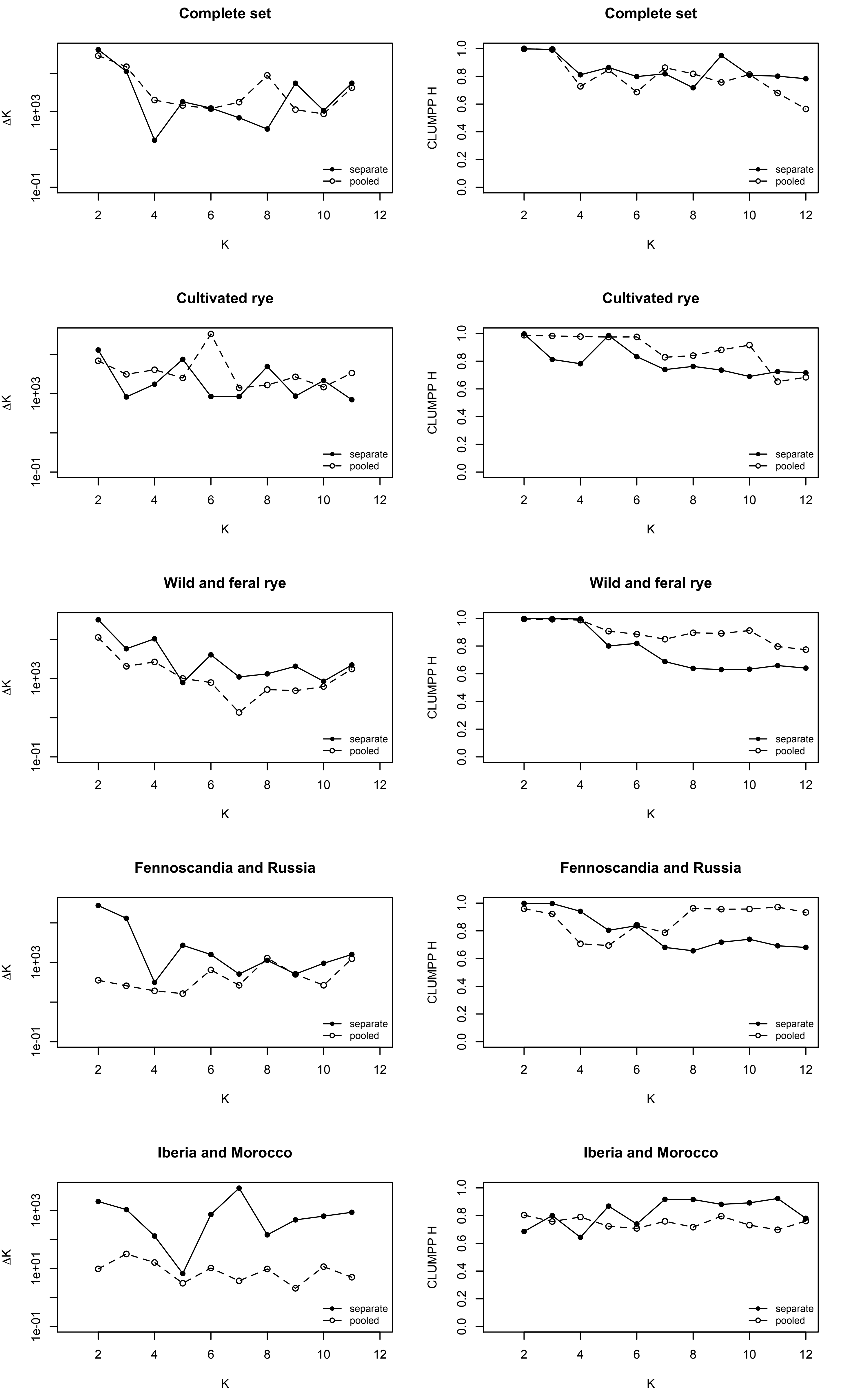
Complete set
ΔK
1e-01
1e+03
separate
pooled
K
2
4
6
8
10
12
Complete set
CLUMPP H
0.0
0.2
0.4
0.6
0.8
1.0
separate
pooled
K
Cultivated rye
ΔK
separate
pooled
K
Cultivated rye
CLUMPP H
separate
pooled
K
Wild and feral rye
ΔK
separate
pooled
K
Wild and feral rye
CLUMPP H
separate
pooled
K
Fennoscandia and Russia
ΔK
separate
pooled
K
Fennoscandia and Russia
CLUMPP H
separate
pooled
K
Iberia and Morocco
ΔK
1e-01
1e+01
1e+03
separate
pooled
K
Iberia and Morocco
CLUMPP H
separate
pooled
K
