# Supplementary material for: Geographical distribution of genetic diversity in Secale landrace and wild accessions
Source: BMC Plant Biol. 2016 Jan 19;16:23. doi: 10.1186/s12870-016-0710-y (PMC4719562; doi:10.1186/s12870-016-0710-y)
Supplement: Additional file 12: — DAPC output based on chloroplast SSRs for K models 2 to 6 for the complete set of accessions. Each bar is one accession averaged on 4 to 6 individuals. Accessions are organized by taxon. A) K = 2, B) K = 3, C) K = 4, D K = 5, E) K = 6 (PDF 11334 kb) [file 12870_2016_710_MOESM12_ESM.pdf]

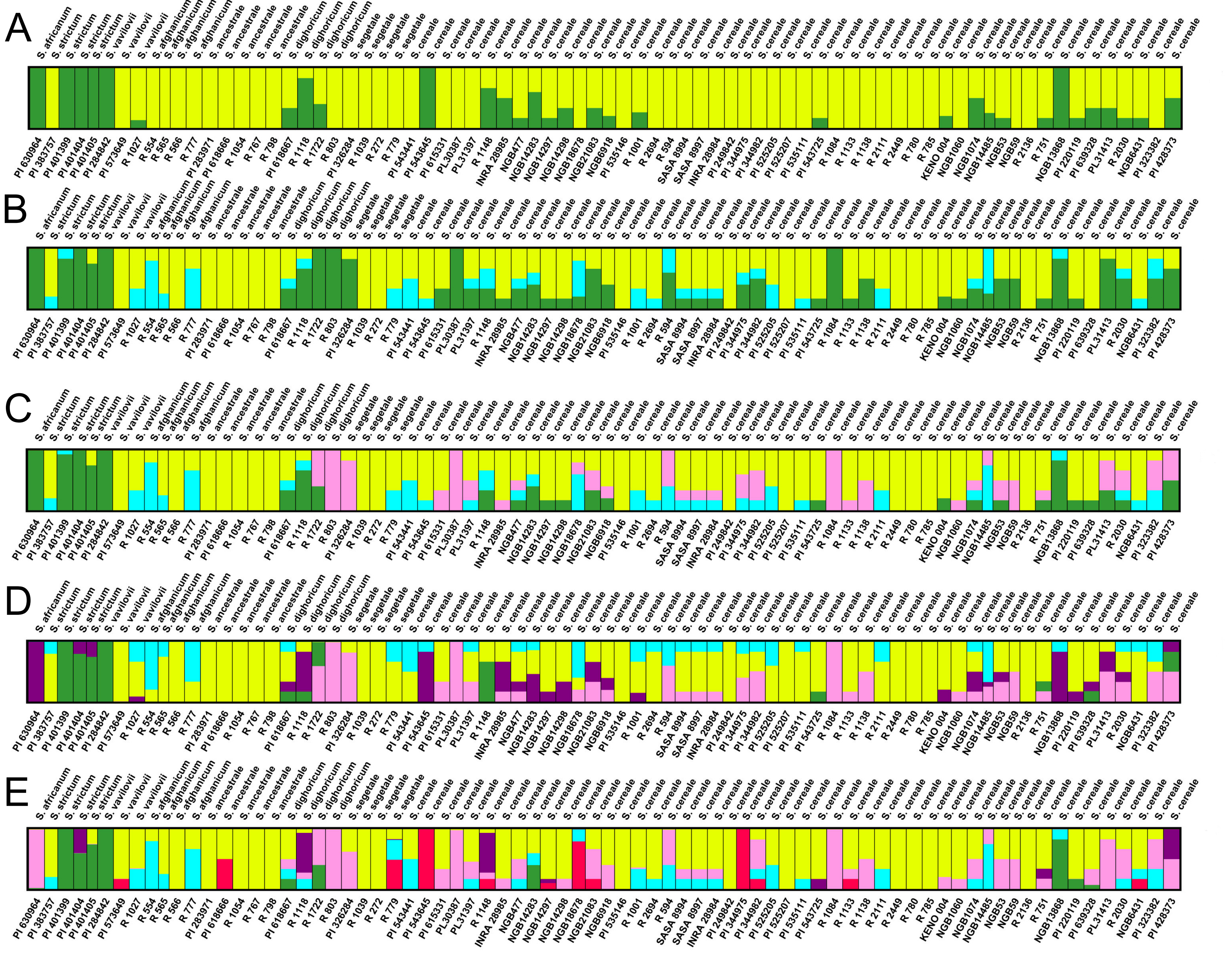

A
B
C
D
E
S. africanum
S. strictum
S. strictum
S. strictum
S. strictum
S. vavilovii
S. vavilovii
S. vavilovii
S. afghanicum
S. afghanicum
S. afghanicum
S. afghanicum
S. ancestrale
S. ancestrale
S. ancestrale
S. ancestrale
S. ancestrale
S. dighoricum
S. dighoricum
S. dighoricum
S. dighoricum
S. segetale
S. segetale
S. segetale
S. segetale
S. cereale
PI 630964
PI 383757
PI 401399
PI 401404
PI 401405
PI 284842
PI 573649
R 1027
R 554
R 565
R 566
R 777
PI 283971
PI 618666
R 1054
R 767
R 798
PI 618667
R 1118
R 1722
R 803
PI 326284
R 1039
R 272
R 779
PI 543441
PI 543645
PI 615331
PL30387
PL31397
R 1148
INRA 28985
NGB477
NGB14283
NGB14297
NGB14298
NGB18678
NGB21083
NGB6918
PI 535146
R 1001
R 2694
R 594
SASA 8994
SASA 8997
INRA 28984
PI 249842
PI 344975
PI 344982
PI 525205
PI 525207
PI 535111
PI 543725
R 1084
R 1133
R 1138
R 2111
R 2449
R 780
R 785
KENO 004
NGB1060
NGB1074
NGB14485
NGB53
NGB59
R 2136
R 751
NGB13868
PI 220119
PI 639328
PL31413
R 2030
NGB6431
PI 323382
PI 428373
